# Orchard monitoring based on unmanned aerial vehicles and image processing by artificial neural networks: a systematic review

**DOI:** 10.3389/fpls.2023.1237695

**Published:** 2023-11-27

**Authors:** Dan Popescu, Loretta Ichim, Florin Stoican

**Affiliations:** Faculty of Automatic Control and Computers, National University of Science and Technology Politehnica Bucharest, Bucharest, Romania

**Keywords:** orchard monitoring, unmanned aerial vehicle, dataset, image processing, neural network, object detection, object segmentation, object classification

## Abstract

Orchard monitoring is a vital direction of scientific research and practical application for increasing fruit production in ecological conditions. Recently, due to the development of technology and the decrease in equipment cost, the use of unmanned aerial vehicles and artificial intelligence algorithms for image acquisition and processing has achieved tremendous progress in orchards monitoring. This paper highlights the new research trends in orchard monitoring, emphasizing neural networks, unmanned aerial vehicles (UAVs), and various concrete applications. For this purpose, papers on complex topics obtained by combining keywords from the field addressed were selected and analyzed. In particular, the review considered papers on the interval 2017-2022 on the use of neural networks (as an important exponent of artificial intelligence in image processing and understanding) and UAVs in orchard monitoring and production evaluation applications. Due to their complexity, the characteristics of UAV trajectories and flights in the orchard area were highlighted. The structure and implementations of the latest neural network systems used in such applications, the databases, the software, and the obtained performances are systematically analyzed. To recommend some suggestions for researchers and end users, the use of the new concepts and their implementations were surveyed in concrete applications, such as a) identification and segmentation of orchards, trees, and crowns; b) detection of tree diseases, harmful insects, and pests; c) evaluation of fruit production, and d) evaluation of development conditions. To show the necessity of this review, in the end, a comparison is made with review articles with a related theme.

## 1 Introduction

The monitoring of modern orchards based on the acquisition and continuous processing of data has become a necessity for obtaining the highest possible production of healthy fruits. Within the data processing field, image processing is of particular interest for orchard monitoring because it efficiently solves several essential aspects like orchard mapping, tree segmentation, production (fruit) evaluation, disease detection, the need for water or special solutions, pest detection, etc. Both RGB (red-green-blue) and multispectral images are used to evaluate the parameters characterizing the orchard problems. They provide a significant volume of information used for efficient monitoring. The correct acquisition of images is necessary so that the regions of interest are of good quality. Various vectors have been used for image acquisition, such as human operators with cameras or smartphones, fixed cameras, cameras on land vehicles, aerial vehicles (autonomous or not), and satellites (Lin et al., 2021). Collecting image data in a complex 3D space, such as an orchard, is a relatively recent challenge made possible by the recent development of new technologies. Consequently, due to both the technological improvements and the economic aspects promoted by large-scale production, many agriculture-related problems have been augmented with the integration of artificial intelligence techniques and remote sensing systems. Although satellites and UAVs (Unmanned Aerial Vehicles) complement each other in the task of inspecting different terrestrial areas, in the case of orchard monitoring, UAVs offer clear advantages such as ultra-resolution, cost-effective operation, increased flexibility for individual tree inspection, and resilience against weather patterns such as cloudy (Alvarez-Vanhard et al., 2021). Not least, for the monitoring of crops in precision agriculture, collaboration with wireless ground sensor networks is of particular importance (Popescu et al., 2020). On the other hand, in complex applications related to orchard monitoring, UAVs have the advantage to take images from either a medium distance (10 m -100 m) through an appropriate design of the trajectories - such as in the case of orchard or tree segmentation (Adamo et al., 2021; Akca and Polat, 2022) or to determine the water stress index (Zhang C. et al., 2021) or from a smaller distance (tens of cm) - such as the case of detecting harmful insects (Aota et al., 2021; Ichim et al., 2022) or fruits (Wang S. et al., 2022). The UAVs compared to terrestrial robots is also a more flexible and less expensive solution. The automatic picking of fruits is an exception. In the future, the use of complex multirobot systems that combine the actions of UAVs, ground robots, and manipulators (Sulistijono et al., 2020; Ju et al., 2022) can lead to an increased degree of automation in modern orchards. However, research papers related to the application of artificial intelligence and the use of drones (UAVs) in the monitoring of orchards are relatively few compared to the monitoring of flat, field crops. This is a consequence of considering the 3D space in orchard applications.

It should not be forgotten that an essential condition for the effective use of UAVs is flights performed beyond the visual range of the operators. Due to the strong increase in the number of operational UAVs, it has become necessary to analyze the conditions for making safe flights in shared airspace. In this sense, working meetings are increasingly taking place at the level of the European Union to update the relevant flight regulations. For the safe operation of many drones, the “U-space” concept was introduced into European legislation (Barrado et al., 2020) to manage UAS (unmanned aerial systems) traffic. It refers to the framework of regulations, technologies, and procedures required to enable safe and efficient drone operations in low-altitude airspace. With the integration of drones into the airspace system, U-space provides a framework for ensuring safety, security, and efficiency in their operation. The continued development and implementation of U-space regulations and technologies are essential to realizing the full potential of drones and their applications in the future. The term refers to a collection of digitized and automated functions and processes aimed at ensuring safe, efficient, and equitable access to airspace for the growing number of civilian drone operators. This is essential for enabling the many benefits of drone technology, such as improved delivery services, monitoring and inspection of agricultural crops, and support for emergency services. Not least, by requiring pilots to obtain a license and submit a flight plan, U-space regulations help to mitigate the risks associated with drone operations and promote the responsible and safe use of this technology.

Efficient monitoring in precision agriculture requires precise mapping of agricultural crops and, implicitly, orchards. That is why the detection and location of orchards and trees in the orchard with the help of aerial robots and neural networks have undergone a spectacular evolution in recent years (Osco et al., 2020; Zhang et al., 2018; Osco et al., 2021). In precision agriculture, terrestrial robots and UAVs were used for instance segmentation and fine detection of crops, trees, and weed plants (Champ et al., 2020; Chen et al., 2019; Khan et al., 2020a). It can be stated that drones and neural networks are essential ingredients in precise and intelligent agriculture. As per (Jensen et al., 2021), pesticide usage is 30% of the total cost in citrus and 42% in olive orchards. The pesticide reduction is discussed in (Özyurt et al., 2022) where UAVs are used to assess areas in need of spraying in a hazelnut. The actual application of pesticides is not straightforward: multi-rotor UAVs are severely restricted in the maximum payload weight. Time is also a factor. (Zortea et al., 2018) show that a month of manual labeling in the field is replaced by a week of manually labeling images obtained from a UAV flight (which may be further reduced to less than a day when automatizing the labeling procedure). Noteworthy, no single algorithm works for any type of orchard/forest (Larsen et al., 2011).

Monitoring of orchards through automated methods based on image processing and artificial intelligence leads to increased productivity while reducing expenses. Application of deep learning for the delineation of visible cadastral boundaries of parcels in rural scenes from UAV imagery can be used with smaller effort for delineation compared to manual delineation (Crommelinck et al., 2019). This means adjusting data processing systems to various conditions, types, or sizes of orchards. Thus, recently, machine learning methods, intelligent classifiers, and, especially, convolutional neural networks (CNN) have been used for the detection, classification, and segmentation of regions of interest (RoI) from images acquired in the orchard for various applications. As a trend, Deep Convolutional Neural Networks (DCNNs) are increasingly used in object detection (Xiao et al., 2020), a particularly important aspect in orchard management (e.g., detection of fruits, diseases, insects, etc.). Deep neural networks and transfer learning were used for food crop identification from UAV images (Chew et al., 2020). In the review paper (Alzubaidi et al., 2021), the main components of DCNN used for object detection are detailed, emphasizing the advantage offered by these networks to automatically detect the main features used without human intervention. Specifically, in fruit detection problems, several recent works have been making use of Deep Learning (DL) methods applied to images acquired at different height levels (Biffi et al., 2021).

The measurement of size, growth, and mortality of individual trees is of utmost importance for orchard or forest monitoring. To this end, the authors (Hu and Li, 2020) proposed a point cloud segmentation method for single trees. They used UAV tilt photography and a simple neural network (NN) for data processing feature extraction and classification tasks with an accuracy of about 90%. A method to detect, geolocate, and identify tree species by UAV flight and NN processing of acquired hyperspectral images is presented by (Miyoshi et al., 2020). UAVs are also used as a cheap and reliable solution for measuring the height of crops (Xie et al., 2021), including orchard trees. In this case, additional spatial information such as the digital terrain model and the ground truth of the height is required. In such cases, it becomes especially important to correct the positioning errors of global navigation satellite systems (GNSS) by different methods. To this end, UAVs are often equipped with a real-time kinematic positioning (RTK) module.

The early detection of tree disease in orchards can significantly improve the control of these diseases and avoid the spread of insects, viruses, or fungi. For example, vine disease detection by automatic methods leads to increase efficiency and productivity of vineyard crops in smart farming, simultaneously with the reduction of pesticides. Therefore, the detection of vine diseases in UAV images using neural networks has been widely addressed recently (Kerkech et al., 2018; Kerkech et al., 2020).

A difficulty that can be encountered in orchard monitoring is the dense tree crowns. This can often cause GPS (Global Positioning System) signal attenuation when the UAV or a terrestrial robot is traveling in an orchard. A method to overcome this drawback is proposed by (Kim et al., 2020) using a CNN to classify patches in the front image in path, tree, or background. For this purpose, the image is traversed successively with sliding investigation windows, and a path score map is generated through the CNN classification results.

**Figure 1:**
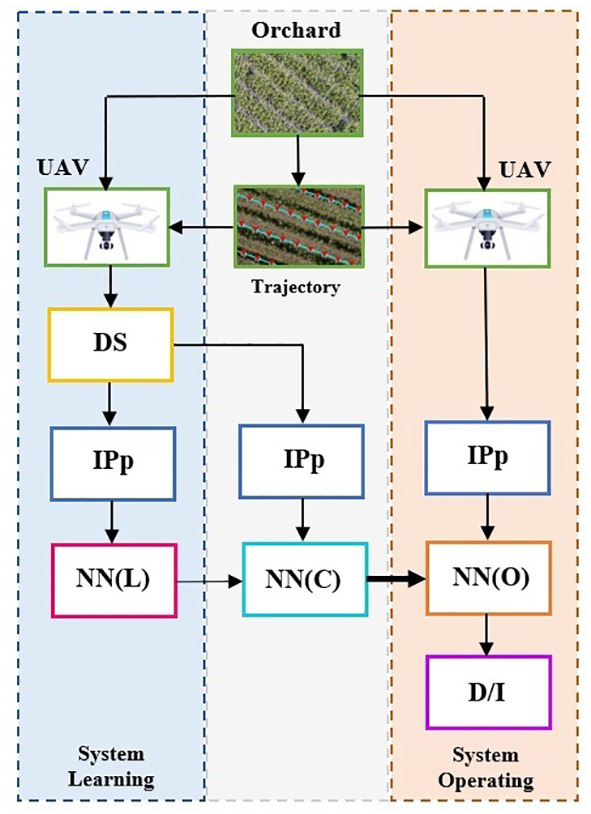
Structure of the orchard monitoring system composed of UAVs and neural networks. UAV – unmanned aerial vehicle (drone), DS – data set, IPp – image preprocessing module, NN(L) neural network learning (parameters and weights), NN(C) – final NN configuration (after validation), NN(O) – neural network implemented for operating phase.

Broadly speaking, an orchard monitoring system based on the use of UAVs and NNs has the structure of **Figure 1**. It has two main paths, system learning and system operating. In the first phase, the UAV acquires the images for the dataset (DS) needed for the learning and validation phases to establish the parameters and weights NN(L). Sometimes the dataset can be a public one. The images need a preprocessing set of operations by IPp (Image Preprocessing module). After learning, validation, and final configuration of structure NN(C), it is implemented in the operating configuration NN(O) on a terrestrial operating station or even on the UAV. The system output is a decision or/and a new image (D/I). In orchard monitoring, the respective applications and images are very different and therefore the choice of UAV trajectories to obtain the most relevant data (images) and especially the choice of NNs used for the analysis of the regions of interest constitute real challenges. Still, newer is the integration of the monitoring of agricultural crops, including orchards, into IoT (Internet of Things). Thus, if real-time processing of monitoring data is required, as in the case of pest detection, a solution presented by (Bhoi et al., 2021) is a UAV assisted by IoT, where images of pests are sent for processing to the Imagga cloud (https://imaga.com), to retrieve the pest information.

The current paper focuses on the importance of UAVs and image processing through artificial intelligence techniques (in particular, CNN) for orchard monitoring from various points of view such as flowering, evolution, diseases, harmful insects, fruit ripening, and picking. Thus, the paper focuses on the new trends in the use of UAVs and image processing based on NNs for efficient monitoring of orchards in precision agriculture with ecological considerations. Apart from the Introduction, the paper contains five sections. Section 2, entitled Survey Methodology, presents the methodology for investigating papers in the field from 2017-2022. Section 3, named UAVs and Cameras Used for Image Acquisition in Orchard Monitoring, presents the UAVs and video/photo cameras used in the analyzed papers, the characteristics of UAV trajectories in orchard monitoring, and develops the aspects related to the design and tracking of UAV trajectories in the orchard. Section 4, Neural Networks Used for Orchard Monitoring, refers to the presentation of the neural networks used, datasets, software, performances, and the new implementation trends based on the fusion of decisions or the combination of several neural networks. Section 5, Applications, is dedicated to the most frequent orchard monitoring applications through the prism of new technologies. In Section 6, Discussions, some observations are made regarding the global aspects of research in the field from the last three years and comparisons with review papers based on the same keywords. The last section is the Conclusions which highlights the important aspects of the paper. All development chapters are accompanied by graphs or synthetic tables. Since there are many notions and definitions that are repeated or are put in tables, in order not to fill unnecessary space and to make it easier to understand, a list with abbreviations is provided as Annex 1.

## 2 Survey methodology

For the systematic review paper, 872 papers were analyzed from different databases such as the Web of Science (311), Scopus (292), and IEEE Xplore (269). Finally, we selected 197 papers (173 research papers and 24 review-type papers) for this review. The eligibility criteria for paper selection were recent publications, new trends in orchard monitoring on different aspects, the impact of contributions, the involvement of UAVs, and the use of NNs in the processing of images acquired in orchards. As the impact, the citations can be a relative criterion because, in general for older papers, the citations are higher than for newer ones. The high rank of publications refers to Category Quartile Q1, Q2, and the Journal Impact Factor in Web of Science 2021. More than 68% of the total references meet this criterion. Most of the papers included in this study are from journals with an impact factor greater than 2. Among the analyzed articles, 167 are from journals and 30 are from conferences. Focusing on a relatively recent period (2017 – 2022), the most representative papers covering the ROI detection, segmentation, and classification in orchard images, using state-of-the-art NNs and UAVs, were investigated. Thus, 184 references between 2017 - 2022, representing 93.40% of the total, were selected, and focusing on 2019 – 2022, as a recent period, 84.69% of references were analyzed. In terms of new trends in using NNs for UAV image analysis, the following directions can be mentioned: a) improvement of a CNN with other networks included in its structure, most often adapted for orchard images, b) systems made up of several CNNs (that can be considered as elements of collective intelligence), and c) systems using CNN combined with other classifiers. This important aspect is detailed in Section 4.

**Figure 2:**
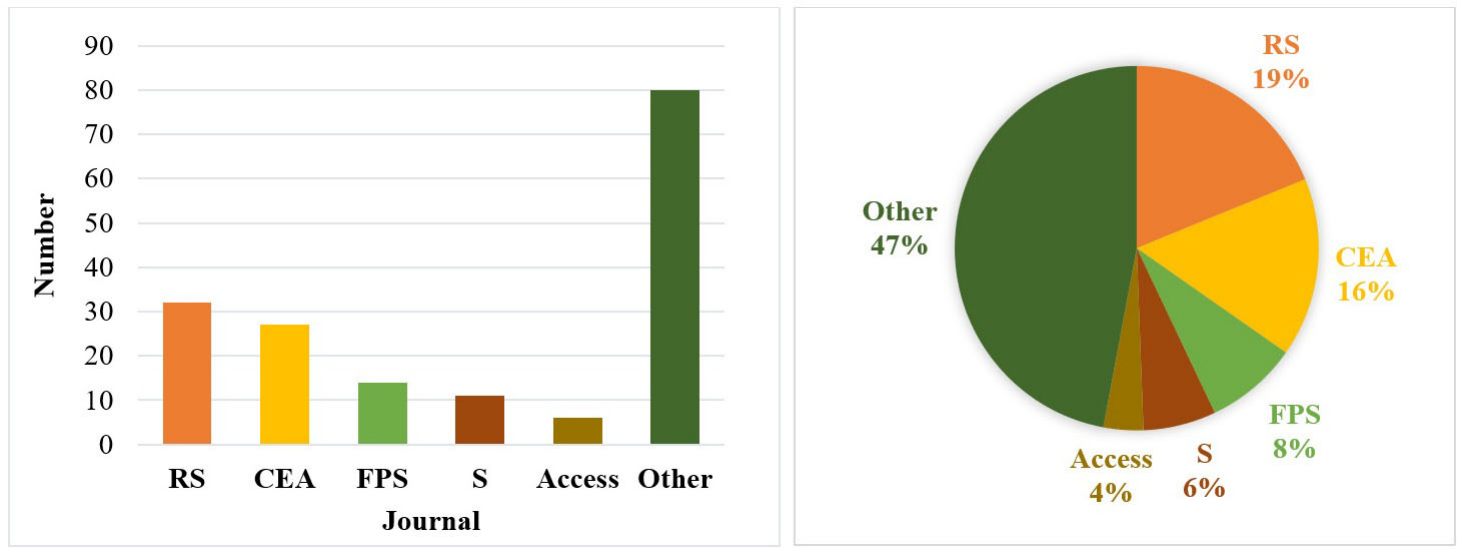
The number (left) and the percentage (right) of papers that are analyzed from journals: Remote Sensing (RS), Computers and Electronics in Agriculture (CEA), Frontiers in Plant Science (FPS), Sensors (S) and IEEE Access (Access).

For the systematic review and meta-analysis, we used a PRISMA (Preferred Reporting Items for Systematic Reviews and Meta-Analyses) (http://www.prisma-statement.org/) flow diagram (**Figure 2**). As can be seen from the diagram, from the total of 892 identified papers, we selected 197 papers according to the criteria mentioned in **Figure 2**. For the paper search strategy, we investigated similar papers in the field. The comparisons and the highlighting of the degree of novelty towards them are underlined in Section 6, Discussions. Most of the analyzed articles were selected from journals (**Figure 2**) such as Remote Sensing (RS), Computers and Electronics in Agriculture (CEA), Frontiers in Plant Science (FPS), Sensors (S), and IEEE Access (Access).

**Figure 3:**
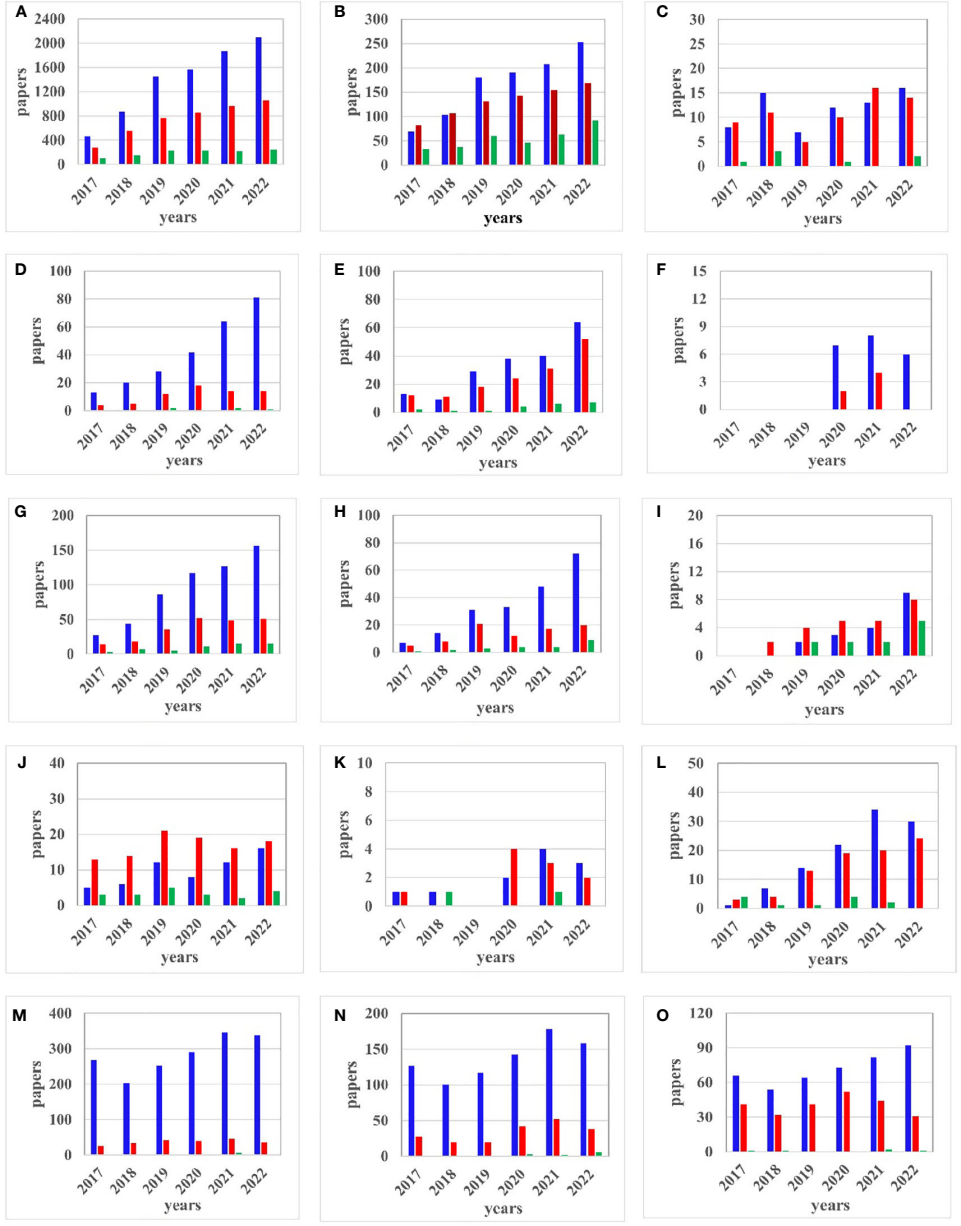
Web of Science-blue, Scopus – red, and IEEE Xplore – green; **(A)** neural networks AND image processing, **(B)** agriculture AND image processing, **(C)** orchard AND image processing, **(D)** orchard AND networks, **(E)** orchard AND uav, **(F)** orchard AND neural networks AND uav, **(G)** auv AND control AND neural networks, **(H)** uav AND trajectory AND neural networks, **(I)** uav AND U-space, **(J)** agriculture AND uav AND image processing, **(K)** orchard AND uav AND image processing, **(L)** agriculture AND uav AND neural networks, **(M)** orchard AND diseases, **(N)** orchard AND insects, and **(O)** orchard AND fruit production.

Although concerns about the orchard, UAVs or NNs used separately are older and the respective fields of study are well-established, the combination of these topics in orchard monitoring is relatively recent. As we considered the new trends in this direction, **Figure 3** is presented our search in Web of Science (blue), Scopus (red), and IEEE Xplore (green) databases (DBs) between 2017 - 2022 considering the following topics: UAV control, UAV trajectory, U-space, agriculture, orchard, NNs, image processing, diseases, insects, and fruit production. It should be noted that to save space in **Figure 3**, the notation “uav” means UAV, UAS, or drone. The search was split between combinations of keywords using the “AND” connector: (A) neural networks AND image processing, (B) agriculture AND image processing, (C) orchard AND image processing, (D) orchard AND neural networks, (E) orchard AND uav, (F) orchard AND neural networks AND uav, (G) uav AND control AND neural networks, (H) uav AND trajectory AND neural networks, (I) uav AND U-space, (J) agriculture AND uav AND image processing, (K) orchard AND uav AND image processing, (L) agriculture AND uav AND neural networks, (M) orchard AND diseases, (N) orchard AND insects, and (O) orchard AND fruit production. The year is labeled on the x-axis and the number of publications identified according to the search in the database is labeled on the y-axis. It can be observed that the increase in research is higher in most of the cases involving NNs and/or UAVs, with an exception in 2022 because of the indexing latency. Furthermore, it should be noted that while we have strived for a fair comparison between Web of Science, Scopus, and IEEE Xplore, they do have different ways to handle queries, such as those we constructed, for obtaining the results from **Figure 3**. Because IEEE Xplore is not a paper database focused on agriculture the number of papers is much smaller compared to Scopus and Web of Science when the topic of agriculture or orchard appears in searches so that they can be neglected. Also, there is a big difference between the number of papers related to the use of NN and/or UAV in orchards compared to agriculture in general. This can be attributed to the difficulties of flying inside the orchards, the consideration of images in depth (tree crowns), and partially covered objects. In general, we see a rapid increase in papers from 2017 to 2022, especially when it comes to NNs and UAVs in orchard monitoring. On the other hand, due to the appearance in 2018 of the legislation regarding U-space, no articles on this topic were published until that year. Likewise, papers considered by us to be important and containing the orchard-UAV-neural network triplet did not appear earlier than 2019.

## 3 UAVs and cameras used for image acquisition in orchard monitoring

UASs including UAVs tend to be the preferred platform for modern orchard monitoring (Zhang C. et al., 2019). UAV is a generic byword for unmanned fixed-wing devices or more usually multi-rotor copters (multicopters). The latter are often quadcopters (with four motors, the minimum number to ensure simultaneous position and yaw angle tracking, hexacopters (six motors), and octocopters (eight motors with redundancy and increased stability). The drawback for the latter is that they are generally more expensive and require expert handling (due to their larger size and increased velocity any improper use may result in property damage and even accidents).

Each platform comes with its own list of, usually complementary, shortcomings. For example, fixed-wing UAVs have significantly more endurance (flight distance) and, sometimes, payload capacity but lack flexibility because they require a minimum speed to avoid a stall and operate at higher heights. They have traditionally been used for photogrammetry, monitoring, spraying, and data acquisition from large areas (Pederi and Cheporniuk, 2015). On the other hand, multicopters have limited battery life (often in the range of 20 - 30 minutes) but can hover in place and may get quite close to the objects of interest (tens of centimeters, at least when safety measures are deactivated). For these reasons, and due to their comparatively low cost, multicopters are the main tool in small and medium-precision agriculture. A comprehensive classification of multicopters cannot be carried out, but they are mostly divided by their number of motors and whether they are commercial (mainly DJI or Parrot variants) or custom-made for a particular research/application project. Currently, the drones most used for crop monitoring, in particular orchards, are medium or small-sized (adequate for image or sensor data acquisition applications). Larger drones are used for spraying, picking, or planting and are not as widespread yet. Lastly, electric multi-rotor drones are the most popular for orchard monitoring applications as the distances traveled are relatively small, and modern batteries have enough autonomy for this kind of application. For a brief enumeration: popular DJI quadcopter variants are Phantom 3 (Horton et al., 2017; Bouroubi et al., 2018; Apolo-Apolo et al., 2020b; Cheng et al., 2020; Fang et al., 2020; García-Murillo et al., 2020; Barbosa et al., 2021; Menshchikov et al., 2021), Mavic 2 Pro (Barmpoutis et al., 2019; Dong et al., 2020; Nguyen et al., 2021), and Inspire 2 (Häni, 2020) for Mavic Pro 3, and (Mu et al., 2018). The authors in (Zortea et al., 2018) use a GYRO-500X4 quadcopter, and (Torres-Sánchez et al., 2018) use a microdrone MD4-1000. Hexacopters such as the Tarot 960 are used by (Nevalainen et al., 2017). For larger payload capacity and increased stability, octocopters have been used in orchard applications (Abdulridha et al., 2019; Ampatzidis et al., 2019; Horstrand et al., 2019; Deng et al., 2020). Arguably, quadcopter models are the most used in orchard monitoring but hexacopters, even if larger and more expensive, are becoming increasingly popular due to propeller redundancy which leads to better stabilization in nominal functioning and increased reliability under hardware loss. A synthetic description of the kinematic and dynamic models of multicopters is given by (Ju et al., 2022).

**Figure 4:**
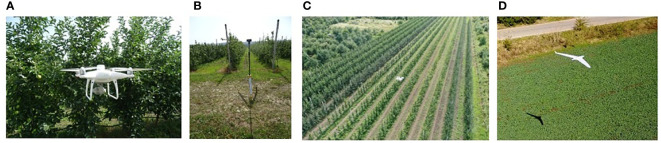
**(A)** Phantom 4 (quadcopter) RTK-flight inside the orchard, **(B)** Fixing the RTK module to the ground, **(C)** Phantom 4 RTK-flight over the orchard, and **(D)** Fixed-wing.

Most commercial UAVs have GPS modules that they use as the go-to positioning system for localization in outdoor settings. The specific difficulties for GPS mainly manifest in cities or other areas with challenging vertical features (the “canyon effect”, where not enough satellites are simultaneously visible for robust localization). In relatively smooth (i.e., of almost constant height) settings such as orchards, GPS in conjunction with sense and avoidance sensors exhibits acceptable performance, with position errors up to 1 m (Nevalainen et al., 2017). A straightforward improvement is the addition of an RTK module (for those drones which support it). This correction mechanism reduces the errors to 2 cm in planimetry and 3 cm in altimetry (Torres-Sánchez et al., 2018). Noteworthy, RTK modules have mostly deprecated the use of physical targets used for GPS correction (visible elements such as AeroPoints (Johansen et al., 2018), whose position is estimated accurately with a GPS module and is later used as a reference in the images taken by the drone. Examples of UAVs like Phantom 4 (quadcopter) with RTK flighting inside the orchard and fixed-wing UAV flighting over the orchard are given in **Figure 4**. The research papers that investigate orchard monitoring based on UAVs with different cameras are presented in the synthetic **Table 1**.

**Table 1:** UAVs with cameras used.

UAV/Type	Camera/Type	References
**▪**DJI Mavic 2 Pro/quadcopter (DJI Corporation)	**▪** Included: Hasselblad L1D-20c, 20MP/RGB	(Barmpoutis et al., 2019; Dong et al., 2020; Nguyen et al., 2021)
**▪**DJI Mavic 3 (DJI Corporation)	**▪** Included	(Häni, 2020)
**▪**Phantom 3 Professional/quadcopter (DJI Corporation)	**▪** Included: RGB, Multispectral 5 channels, 12 MP	(Horton et al., 2017; Bouroubi et al., 2018; Apolo-Apolo et al., 2020b; Cheng et al., 2020; Fang et al., 2020; García-Murillo et al., 2020; Barbosa et al., 2021; Menshchikov et al., 2021)
**▪**Phantom 4, 4 PRO, 4 RTK/ Quadcopter (DJI Corporation)	**▪**Included: RGB, Multispectral 5 channels, 12 MP	(Lobo Torres et al., 2020; Fuentes-Pacheco et al., 2019; Ampatzidis et al., 2020; Apolo-Apolo et al., 2020a; Gallardo-Salazar and Pompa-García, 2020; Kalantar et al., 2020; Schiefer et al., 2020; Yang, M.-D. et al., 2020; Nguyen et al., 2021)
**▪**DJI Matrice 100/quadcopter (DJI Corporation)	**▪**Different: Logitech C310 webcam, MicaSense RedEdge-M/multispectral	(Hulens et al., 2017; La Rosa et al., 2020; Sarabia et al., 2020)
**▪**DJI Matrice 210/quadcopter/possible RTK (DJI Corporation)	**▪** Different: Two cameras/RGB -48 MP (Sony Alpha 7) and multispectral 4 channels (Parrot Sequoia)	(Ampatzidis et al., 2020; Jurado et al., 2020)
**▪**4HSE-EVO/quadcopter (ITALDRON)	**▪** MicaSense RedEdge-M/multispectral	(Adamo et al., 2021)
**▪**DJI Inspire 1/Quadcopter (DJI Corporation)	**▪**Included: RGB	(Hu and Li, 2020)
**▪**DJI Inspire 2/Quadcopter (DJI Corporation)	**▪**Included: RGB	(Mu et al., 2018)
**▪**DJI Matrice 600/hexacopter/possible RTK (DJI Corporation)	**▪** Different: Zenmuse, Specim FX10, added/Multispectral 5 channels, Resonon Pika L 2.4 hyperspectral, MicaSense RedEdge-M/multispectral	(Abdulridha et al., 2019; Ampatzidis et al., 2019; Horstrand et al., 2019; Deng et al., 2020)
**▪**OktoXL 6S12/octocopter (Mikrokopter)	**▪**Alpha 7R, Sony/RGB	(Schiefer et al., 2020)
**▪**eBee Sense Fly/fixed wing (MikroKopter GmbH)	**▪**Different: Parrot SEQUOIA, Multispectral 4 channels, senseFly S.O.D.A.	(Duarte et al., 2020; Schoofs et al., 2020)
**▪**Trimble UX5 fixed wing (Trimble.Applanix)	**▪**Different: RGB and multiple bands	(Adhikari et al., 2021)

We observe a large variety of cameras and related applications. Although UAVs can be equipped with payloads containing various types of image or video sensors (RGB cameras, multispectral, hyperspectral, thermal, SAR), in orchard monitoring applications the most used are RGB and multispectral (**Table 1**). Many applications in crop monitoring use small UAVs with included video/photo cameras, without the possibility of attaching other cameras. In the case of larger UAVs, there is the possibility of using different cameras, depending on the requirements. Even if the number and type of UAVs are relatively limited, there is a great variation in the types and numbers of payloads with thermal (Mesas-Carrascosa et al., 2018; Pádua et al., 2020), multispectral (Horton et al., 2017), video (Torres-Sánchez et al., 2018) cameras, or even spectrometers (Ocean Optics (Nevalainen et al., 2017). Relatively recently, cameras with integrated machine learning features have started to appear in UAV applications due to reductions in cost, energy requirements, and weight.

### 3.1 Characteristics of UAV trajectories in orchard monitoring

For orchard monitoring, the UAV trajectory can be a challenge, because in many applications it can be a 3D trajectory, above and inside the orchard. For a programmed, automatic flight, the lateral distance from the crown of the trees correlated with the protection devices of the UAV creates difficulties in establishing and following the trajectory. Regardless of the trajectory specifics, some parameters are important. Among the most popular are total trajectory time, ground velocity, and flight altitude. As mentioned in (Torres-Sánchez et al., 2018) run times may be significant for terrestrial platforms with respect to UAV limitations. They give the example of an almond orchard where 6.2 km was covered in 1.5 hours (with multiple passes). In general, the UAV velocity is higher compared to ground-based vehicles. (Cheng et al., 2020) gives 5 m/sec for the UAV flight whereas (Dong et al., 2020) runs the UAV at 3 m/sec, and (Mu et al., 2018) consider a speed of 2.5 m/sec. Altitude is also a factor and it may vary significantly, depending on mission specifics: (Dong et al., 2020) mentions 50 m, (Mesas-Carrascosa et al., 2018) 120 m, and (Mu et al., 2018) 30 m. These altitude values are for top-down observations (photogrammetry missions or disease/humidity monitoring). Flying close to the treetops or even in between tree rows obviously reduces the flight height to 1 m - 5 m. In this context, noteworthy elements which characterize an orchard are row inter and intra-distance. These depend on the type of tree and even on the country. (Cheng et al., 2020) reports 4 m between trees and 5 m between rows in the case of cherry trees and 3 m and 4 m respectively for apple trees. (Dong et al., 2020) mentions spacings of 4 m and 1.5 m (apples) and 4.5 m and 2 m (pears).

Beyond economic or availability factors, various mission specifics may force a particular choice of UAVs. Small/convoluted domains may require aggressive maneuvering which, for fixed-wing UAVs, is very difficult. On the other hand, large fields may lead to battery depletion. This is a major issue since typically a battery takes significantly more time to charge than to discharge. A typical solution is swapping the battery frequently for increased flight duration (a stop where the battery is quickly changed with a full one and the flight is then resumed). These considerations directly influence the choice of trajectory and mission parameters.

Another aspect is the flexibility of the trajectory. The more common approach is to pre-compute the trajectory, couple it with an autonomous sense-and-avoidance system, and then passively track the experiment (the supervisor intervenes only when urgency stop commands are required). Note that typical sense and avoidance mechanisms impose a hard limit of 1 m - 2 m between the drone and possible obstacles. A simple solution can be to adapt the avoidance mechanism and make sure at the supervision level that the drone trajectories accurately avoid the obstacles (tree branches) *via* embedded cameras or RTK-corrected GPS localization.

Not least, and especially for small and medium-sized drones, the presence of wind is a major factor. Thus, flights are often scheduled in periods when the wind is at a minimum. Less common, but still present is the case where flights are determined by the mission particularities. For example, some harmful insects (HH) have a daily cycle which means that they are active (and hence visible) only in the early morning and in periods of reproduction (Leskey and Nielsen, 2018).

While the more interesting missions are those closer to the ground, the most common are still the photogrammetry missions. While conceptually simple, the output of such as mission may be significantly affected by various flight parameters. Beyond those related to resolution (fly height, camera specifications) and mosaic/3D reconstruction (front and sideways overlapping for consecutive images), flight direction, solar irradiation, camera inclination, and whether the pictures are taken time or position-wise, are also relevant (Tu et al., 2020). Thus, most orchard applications reduce to a coverage problem. Beyond the technicalities imposed by the particularities of the problem (Mokrane et al., 2019) enumerate the generic properties that the resulted trajectory must verify: i) cover all points of interest; ii) avoid overlapping routes; iii) avoid obstacles; iv) as much as possible, use simple primitives to construct the trajectory (straight lines and/or arcs of circles).

Most users do not have the knowledge or the desire to design from zero a trajectory generator. There are various local or cloud-based applications that permit interaction with a drone. We may classify these apps depending on the level to which they interact/supersede the original architecture of the drone. Many of them reduce to providing an ergonomic interface that allows defining various simple missions like following waypoints, covering an area with straight parallel lines, etc. It is more challenging to intervene in the actual control scheme and provide direct control actions. For example, in (Horton et al., 2017) the cloud based DroneDeploy is used to construct a flight plan, by interfacing with both GoogleMaps and the drone. Extremely common is the Pixhawk+Ardupilot autopilot controller. This implements all low-level control actions leaving to operator only the task of providing the list of waypoints. Pix4dmapper was used in (Mesas-Carrascosa et al., 2018; Pádua et al., 2020) to triangulate and mosaic the images. (Jensen et al., 2021) uses MoveIt for 3D motion planning and the octomap_mapping package for 3D occupancy grid mapping. ODM (Open Drone Map - https://github.com/OpenDroneMap), in its multiple ports, is an open-source effort that aims to cover the entire workflow of image post-processing for photogrammetry applications.

As stated in the introductory section, due to the increase in the number of drones and flight areas, it is necessary to establish and update relevant flight regulations for UAVs. In Europe, the U-space concept has been formalized through the European Union’s U-space Regulation, which was adopted in 2019 and came into effect in 2021. The regulation provides requirements for the design, implementation, and operation of U-space services, including registration and identification of drones, communication protocols, and geo-fencing. The unmanned aircraft system traffic management (UTM) concept is also being developed in other parts of the world (United States), with a range of different approaches being taken. It is safe to say that, in one form or another, a framework of rules and regulations has already taken shape and will govern human-UAV interactions in the future.

### 3.2 Trajectory design

For most orchard-related missions, the drone does a top-down analysis where the camera is oriented downwards to take pictures while the drone flies in a plane parallel with the horizontal one and at an altitude that is both safe and balances coverage and image resolution. (Ronchetti et al., 2020) provides a list of common altitude values. (Johansen et al., 2018) carries an interesting analysis of tree detection (center position and canopy delineation) in a lychee orchard by changing the height at which the pictures are taken. This is done to find a balance between coverage speed and precision of the estimates. Worth mentioning is that photogrammetry applications usually take photos at a constant sampling time (as a proxy for equal distances between coordinates). Thus, it is important to maintain a constant ground velocity along the flight path. This must be a design requirement at the trajectory generation step and must also be enforced by feedback laws due to the presence of various disturbances. The goal of such missions is often along the lines of photogrammetry in the sense that partially overlapped images are merged (offline, in a computationally intensive effort) into a large-scale map from which various features of interest are extracted. For example, (Torres-Sánchez et al., 2018) estimate the shape of the tree. Crown volume estimation is carried out by (Torres-Sánchez et al., 2015). Noteworthy, in the latter, the authors mention a root mean square error of 0.39 m for tree height estimation. This may be interpreted as a safety factor for tree-level flights.

One of the few results which explicitly mentions flying at tree level is (Jensen et al., 2021) which implements a three-step run: first, a map of the orchard is created by flying over; second, rows and trees are identified from the acquired images; third, the drone tracks a trajectory between trees. The caveat is that the algorithm was only tested in simulation (within the ROS/Gazebo framework).

From papers that illustrate actual experiments various practical interactions among the UAV components also emerge. For example, (Mesas-Carrascosa et al., 2018) carries out a photogrammetry path planning (straight parallel lines) with emphasis on flight duration due to the need of calibrating the thermal sensors (there is drifting proportional to the duration of the flight). (Mesas-Carrascosa et al., 2018) also proposes to avoid the pre-calibration step by doing it post-flight over the images themselves and by carrying an in-flight drift correction for microbolometer thermal sensors.

Of course, the most important element for rotary drones is battery life. Their increased flexibility comes at the price of significantly less autonomy than in the case of fixed-wing UAVs. Hence, energy efficiency is paramount in trajectory design and influences mission planning at all stages. This may mean proposing very simple trajectories: straight lines as in (Mesas-Carrascosa et al., 2018) or a grid pattern as in (Mu et al., 2018). Usually, the UAV dynamics are ignored when assessing battery consumption (Furchì et al., 2022). Still, the drone behavior and type of trajectories employed can have a disproportionate effect on battery life. (Pradeep et al., 2018) provides a first principles approach to quantify consumption for the climb, cruise, and descent phases (with application to a DJI Phantom 4 quadcopter).

From a dynamics viewpoint “trajectory” means that both position and attitude must be specified at each moment of time during the flight. Except for laboratory/experimental setups, this is hidden by the embedded control software of the drone. Rather, the end-user simply gives a list of waypoints from which the drone’s control mechanism designs a suitable trajectory. Choosing the waypoints that define a path is quite challenging, depending on the mission complexity. In such cases, often heuristic and graph-based methods are employed. For example, (Ochoa and Guo, 2019) combine a genetic algorithm (to determine way-point locations) with the Dijkstra algorithm (for path construction).

Many times, there are multiple flights carried during the same mission. Often, the first flight is for sensor calibration, an update of position information, and an update of the environment’s map (new features of interest, changes in positioning, etc.). Only in the subsequent step, the actual flight (the one where data is gathered) is done. Thus, a typical workflow is as the one from (Horstrand et al., 2019):

- initial flight to assess the environment,
- planning step on the flight management system (choose waypoints, area of interest, etc.),
- start the way-point tracking and supervise the UAV during its flight, with the possibility to update path/sending “turn to base” commands.

In the case of modern orchards, for UAV navigation inside the orchard, among the rows of trees, the orchard can be modeled as an aisle graph (Sorbelli et al., 2022) so that the images are collected as efficiently as possible. In this case, it is about collecting images to detect some harmful insects on trees. Most if not all graph-based methods are based on variations of the Traveling Salesman Problem (TSP). (Furchì et al., 2022) uses a Steiner TSP implementation where only a subset of the nodes must be visited. The paper is also noteworthy for considering battery usage and integrating it as a weight for the graph edges.

In general, formulating decision problems (graph-based or otherwise) for efficient orchard travel leads to a difficult optimization problem. Authors (Furchì et al., 2022) provided a mixed-integer formulation that makes use of binary variables to characterize decisions in the problem (which node is next, which path is followed from a given list, etc.). Such methods are prone to numerical issues and quickly become impractical for real-time implementations. The usual approach is then to simplify the problem and solve it to a sub-optimal solution. In this case, the computation time reduction is significant and the loss in performance is negligible. The heuristic methods employed are usually based on evolutionary procedures or greedy algorithms.

### 3.3 Trajectory tracking

Most agricultural UAV applications give the trajectory as a list of waypoints with associated actions. For example, the API (programming interface) of DJI drones allows by default to give a list of up to 100 waypoints and to associate up to 15 actions for each of them (camera focus, take an image, start/stop the video, etc.). The actual trajectory (path and input actions) is computed onboard the UAV by the autopilot. At this level, further restrictions may be considered (from the sensor and avoidance module, limitations on control actuation, etc.) which will affect the path’s shape. Lower-level interactions are usually relegated to experimental drones used in research laboratories (Parrot Mambo or Crazyflie nano-drone, NXP HoverGames for mid-sized drones, etc.).

Any path-tracking algorithm is as good as the quality of information that it receives (Li, J.-M. et al., 2021). Usually, GPS (possibly corrected by an RTK module) information is employed. Albeit ubiquitous in recent years, GPS may be replaced or supplanted by other approaches. (Emmi et al., 2021) fusions information from 2D Lidar and RGB cameras to identify key locations and working areas which are next integrated into a semantic layer where the various features of interest have certain types (lane, alley, etc.) among which the UAV transitions. The authors in (Stefas et al., 2016) present a vision-based approach for UAV navigation within an orchard. Both the monocular and binocular cases are analyzed. For the former, additional information about the structure of the orchard rows is used and for the latter, a depth-perception algorithm is implemented. In (Hulens et al., 2017) the vision-based approach also makes use of the orchard characteristics: the feasible path is determined by first detecting the center and end (the vanishing point) of the current corridor.

## 4 Neural networks used for orchard monitoring

The use of artificial intelligence and especially NNs for image processing in various fields of agriculture has led to a significant improvement in performance in tasks of detection, segmentation, and classification of regions or objects of interest. Thus, from the investigated researched papers, an improvement in orchard monitoring performances can be noted by NNs in the processing of orchard images. From **Figure 3** it can see that the number of research papers that study the use of NN in orchard monitoring increased in the interval 2017-2022. Most of the NNs in the analyzed papers in this study used RGB images and few multispectral images as in (Kerkech et al., 2020).

### 4.1 Series of neural networks and their representants for image processing in orchard monitoring

Because orchard monitoring involves high-level image processing functions in various conditions, the NNs used in orchard monitoring for image processing were very diverse. Most often, the classification can be used for a special segmentation based on pixel classification named semantic segmentation. The name of the used NNs is explained in the list of abbreviations (Annex 1). The NNs for object detection, classification, and segmentation functions (including semantic segmentation) used in the investigated references are presented in **Table 2**. In some applications, the NNs from popular series, having small structural changes, got the names of respective applications like VddNet - Vine Disease Detection Network (Kerkech et al., 2020) and MangoYOLO (Koirala et al., 2019a)

**Table 2:** NNs used in orchard monitoring (C, classification; D, Detection; S, segmentation or semantic segmentation).

NN series	Representatives/configuration	Function	References
**▪CNN**	**▪**CNN simple	**▪**C	(Kestur et al., 2019; (Kim et al., 2020; Li, Y. et al., 2020; (Csillik et al., 2018; Zortea et al., 2018; Lei et al., 2022)
	**▪**Multi-layer perceptron	**▪**D	(Nevalainen et al., 2017; Fernandez-Gallego et al., 2018)
	**▪**Sandglass bottleneck	**▪**C	(Chen, T et al., 2021)
	FCN	**▪**S	(Marmanis et al., 2016; Osco et al., 2021)
	**▪**CaffeNet	**▪**C	(Bouroubi et al., 2018)
**▪DaSNet**	**▪**DaSNet-A, DaSNet-B, DaSNet-C, DaSNet-v2	**▪**D, S	(Kang and Chen, 2019; Kang and Chen, 2020a)
**▪DeepLab**	**▪**DeepLab-ResNet	**▪**D, S	(Dias et al., 2018)
	**▪**Deep-LabV3 +	**▪**S	(Osco et al., 2021; Li, D. et al., 2022; Zhang X. et al., 2021)
**▪DensNet**	**▪**DensNet 121	**▪**D, C	(Nguyen et al., 2021; Peng et al., 2023)
**▪Encoder - Decoder**	**▪**CED-Net	**▪**D	(Kerkech et al., 2020)
	**▪**Spatial Pyramid- oriented Encoder-Decoder Cascade CNN	**▪**S	(Yuan and Choi, 2021)
	Staked Autoencoder	**▪**D	(Deng et al., 2020)
	**▪**VddNet with three autoencoders (Vine Disease Detection Network)	**▪**D	(Kerkech et al., 2020)
**▪FCRN**	**▪**FCRN	**▪**D	(La Rosa et al., 2020)
**▪GoogLeNet**	**▪** Inception modules	**▪**C	(Breslla et al., 2020)
**▪HRNet**	**▪**HRNet	**▪**D, C, S	(Biffi et al., 2021)
**▪Inception**	**▪**Inception v3	**▪**C	(Fang et al., 2020; Hansen et al., 2020; Zhang, H. et al., 2019)
**▪LeNet**	**▪**LeNet5	**▪**C	(Kerkech et al., 2018; Kerkech et al., 2020)
**▪LedNet**	**▪**LedNet	**▪**S	(Kang and Chen, 2020b)
**▪RBF**	**▪**RBF/RBF+KNN	**▪**D	(Fernandez-Gallego et al., 2018; Abdulridha et al., 2019)
**▪R-CNN**	**▪**R-CNN	**▪**D	(Zhang et al., 2018; Biffi et al., 2021)
	**▪**Faster R-CNN	**▪**D	(Ren et al., 2017; Apolo-Apolo et al., 2020a; Apolo-Apolo et al., 2020b; Biffi et al., 2021; Barmpoutis et al., 2019; Cunha et al., 2021; Khan et al., 2021 Deng et al., 2022; Hu et al., 2022)
	**▪**Mask R-CNN	**▪**D, S	(He et al., 2017; Barmpoutis et al., 2019; Jia et al., 2020; Machefer et al., 2020; Santos et al., 2020; Iqbal et al., 2021; Zhang, W. et al., 2022)
	**▪**Libra R-CNN	**▪**D	(Biffi et al., 2021)
	**▪**Cascade R-CNN	**▪**D	(Biffi et al., 2021)
**▪ResNet**	**▪**ResNet 18	**▪**C	(Zhang et al., 2021; Zhang, X. et al., 2019)
	**▪**ResNet 50	**▪**C	(Fang et al., 2020; Park et al., 2020; Nguyen et al., 2021)
**▪RetinaNet**	**▪**RetinaNet	**▪**D	(Culman et al., 2020)
**▪SegNet**	**▪**SegNet	**▪**S	(Fuentes-Pacheco et al., 2019; Ochoa and Guo, 2019; Majeed et al., 2020; Menshchikov et al., 2021; Osco et al., 2021)
**▪SqeezeNet**	**▪**SqeezeNet	**▪**C	(Park et al., 2020; Nguyen et al., 2021)
**▪SSD**	**▪**SSD	**▪**D	(Aota et al., 2021)
	**▪**SSD with FSAF module	**▪**D	(Biffi et al., 2021)
**▪UNet**	**▪**Simple UNet	**▪**D, S	(Oliveira et al., 2019; Lin and Guo, 2020; Menshchikov et al., 2021; Osco et al., 2021)
	**▪**UNet with SE-ResNeXt-50 as encoder	**▪**S	(Liu et al., 2021; Shang et al., 2021)**/**
	**▪**UNet with VGG-16 encoder	**▪**D, C, S	(Fawakherji et al., 2019; Kattenborn et al., 2019)
**▪VGG**	**▪**VGG16	**▪**C	(Park et al., 2020; Nguyen et al., 2021)
	**▪**VGG19	**▪**C	(Fang et al., 2020; Miyoshi et al., 2020)
**▪Xception**	**▪**Xception	**▪**C	(Fang et al., 2020)
**▪YOLO**	**▪**YOLOv2/improved	**▪**D	(Santos et al., 2020)
	**▪**YOLOv3/improved	**▪**D	(Ampatzidis et al., 2019; Li, J.M. et al., 2021; Liu and Wang, 2020; Santos et al., 2020; Chen, C.J. et al., 2021),
	**▪**YOLOv3/Tiny	**▪**D	(Chen, C.J. et al., 2021)
	**▪**YOLOv4	**▪**D	(He et al., 2020; Li D. et al., 2021; Lin et al., 2022; Popescu et al., 2022b)
	**▪**YOLOv5	**▪**D	(Li, D. et al., 2022; Lyu et al., 2022)
	**▪**YOLO BP	**▪**D	(Zheng et al., 2021)
	**▪**YOLOF-snake/ResNet101 as backbone	**▪**D, S	(Jia et al., 2022)
	**▪**YOLOX	**▪**D	(Zhang, Y. et al., 2022)
	**▪**YOLOP	**▪**D	(Sun et al., 2023)

**Figure 5:**
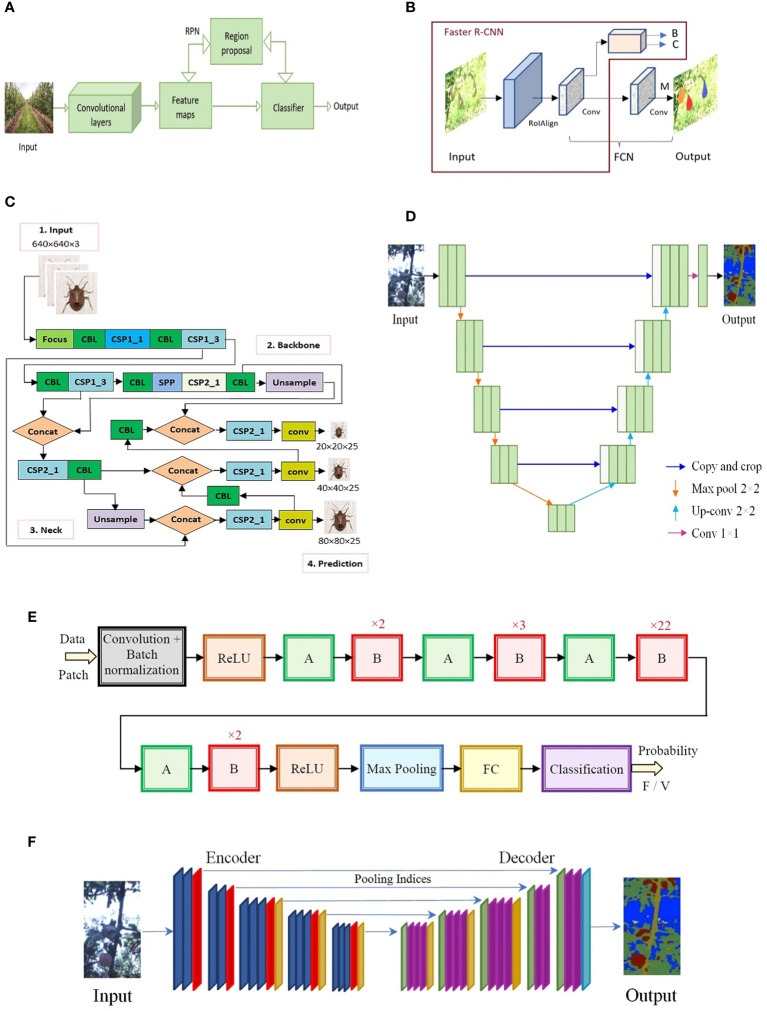
**(A)** Faster R-CNN, **(B)** Mask R-CNN (B-box bounding, C-class, M-mask), **(C)** YOLO v5 (Popescu et al., 2022), **(D)** U-Net architecture, **(E)** ResNet (Ichim and Popescu, 2020), **(F)** Segnet.

The most used NNs were those from series R-CNN (Region-Based CNN) (Girshick et al., 2014), YOLO (You Only Look Once) (Redmon et al., 2016), U-Net (Ronneberger et al., 2015), ResNet (Residual Neural Network) (He et al., 2016), and SegNet (Semantic Segmentation Network) (Badrinarayanan et al., 2017). The basic structures of these important series are given in **Figure 5**. Among them, the YOLO-type NNs had the greatest growth trend. Details regarding the architectures and layers of the most used NNs in image processing for object detection, classification, and segmentation are given by (Alzubaidi et al., 2021; Bhatt et al., 2021). An interesting review (Nawaz et al., 2022) presents the detection of objects in multimedia using NNs, considering single-stage detection and two-stage detection algorithms. The advantages and disadvantages related to precision, complexity, and speed of operation, in various applications such as object detection, multi-object detection, and real-time object detection, were highlighted. The analyzed networks (proposed until 2020) were those from the YOLO, SSD, and RetinaNet series, for the single-stage algorithm, and R-CNN for the two-stage algorithm. Representatives from these series can also be found in the references analyzed in this paper.

R-CNN which is based on a two-stage algorithm for object detection has two important representatives: Faster R-CNN (Ronneberger et al., 2015) and Mask R-CNN (He et al., 2017) which share significant commonalities. Faster R-CNN provides two pieces of information for each candidate object, the classification (class label) and the bounding box (regression). Mask R-CNN extends Faster R-CNN by providing three pieces of information at the output: the class (C), the bounding box (B), and the segmentation mask (M) for each selected region of interest. For the latter, a branch (pixel-to-pixel alignment) is added in parallel in the Faster R-CNN structure. Since this branch has reduced additional computational effort, the network remains quite fast.

Both Faster R-CNN and YOLO are detection networks with object detection accuracy between 63.4% and 70% (Diwan et al., 2022). The YOLO series including several variants (like YOLO v1, v2, v3, v4, v5, v6, v7, v8, YOLOX, etc.) are networks in one stage, and for this reason, they are much faster than Fast R-CNN or Faster R-CNN which are detectors in two stages. Object detection in this case is seen as a regression problem and not a classification one. The areas of interest (objects) are identified, and their positioning is established by a bounding box associated with the probability of belonging to a class.

Faster R-CNN (**Figure 5A**) is a two-stage object detection algorithm providing the bounding box and classification. It can be successfully used for fruit detection in the natural environment in difficult conditions and positions (leaf occlusion, fruit occlusion, fruits in shadow, and different light exposure). A challenge in fruit detection is the great number of fruits (sometimes overlapping) in an input image. Also, it can be used for the detection of diseases and insect pests on fruits.

Mask R-CNN (**Figure 5B**) is like Faster R-CNN and adds to the output a binary mask for segmentation of the detected object. It gets the region where the fruit is located. It can detect and segment fruits in the natural environment (apples, pears, citrus, logan fruit bunches, etc.) in difficult conditions and positions. It was used for the identification and segmentation of trees in orchards from aerial imagery (orthophoto maps).

YOLO is a single-stage object detection algorithm providing the bounding box and classification. It is composed of four sections – input, backbone, neck, and prediction – which allow the detection and localization of objects of different sizes (including small objects) in orchards, like fruits, flower clusters, and insects. It can detect and identify fruits in the natural environment (apples, pears, citrus, logan fruit bunches, etc.) in difficult conditions and positions (covered by leaves, fruits in shadow, fruits at different distances from the camera, and fruit cluster) with precise box location and high accuracy. The various variants of YOLO networks consider a compromise between speed, accuracy, and simplicity. Many of them can be implemented directly on the UAV, for real-time applications simultaneously with video acquisition. The structure of the well-known YOLO v5 is presented in **Figure 5C**.

U-Net (Ronneberger et al., 2015) series is especially important in image segmentation. Although U-Net networks have good segmentation accuracy, they can be trained with relatively few images. In a classic way, the network architecture is made up of two paths (subnets), the first one is contraction type (encoder) and the second one is expansion type (decoder). At each level of the two paths, there are concatenations (skip connections) between the up-sampling of the feature map and the corresponding down-sampling of the feature map. In the new improved versions of the network, various NNs are placed on the encoder as blocks instead of the original ones. Examples of such improved U-Net are given by (Bhatnagar et al., 2020), having ResNet 50 as a backbone, and (Liu et al., 2020), having SE-ResNeXt 50 as a backbone. The basic U-net architecture is presented in **Figure 5D**. Variants of U-Net were used in important applications like the segmentation of trees in the orchard and collecting orchard environment information from UAV images, segmentation of plantation cover area, segmentation of diseased plants and pests, and mapping of the tree species.

ResNet, the winner of the ILSVRC 2015 competition (He et al., 2016), introduced the elements of shortcut connections, within layers providing multi-layer connectivity. As a result, it has a lower computational complexity. Depending on the number of layers ResNet has more representatives: ResNet 18, ResNet 34, ResNet 50, ResNet 101, ResNet 110, ResNet 152, ResNet 164, and ResNet 1202. The most used type in the investigated papers was ResNet50 containing 49 convolutional layers and one FC layer (Alzubaidi et al., 2021). For example, the ResNet network from **Figure 5E** (Ichim and Popescu, 2020) was used to detect flooded zones in an area with vegetation (crops), the meaning of the notations (to save space) being the following: A and B— skip connections, repetitive modules, FC—fully connected layer, F—flood type patch, V—vegetation type patch, and n—number of module repetition). The image was partitioned into patches according to a specific algorithm and each patch (of small size) was classified/segmented as being flood or vegetation. This decomposition into patches can also be used to detect small objects (e.g., insects) compared to the whole image.

The SegNet network (**Figure 5F**) introduced in 2015 (Badrinarayanan et al., 2017) is like an encoder-decoder structure that, in the final stage, has a pixel-wise classification layer. Each layer in the encoder has a corresponding layer in the decoder. Finally, the multi-class soft-max classifier provides for each pixel a probability of belonging to a class, being thus possible a semantic segmentation of the regions of interest (RoIs). It was used in applications like tree localization and classification from aerial imagery, estimation of trees density (large-scale orchard monitoring), segmentation of trunks, branches, and trellis wires (orchard of trees on trellis wires).

As we mentioned, when the databases were unbalanced or the images collected from the orchards were insufficient, some authors used data augmentation techniques such as translations, rotations, transposition, rescaling, reflections, or changing the intensities on color channels. Usually, techniques for image preprocessing, size reduction, or cropping smaller windows were also used before entering the NNs.

In many applications, it has been proven that deep CNNs (DCNNs) can learn the invariant representations of images (as in the case of supervised learning) and can achieve performance at the level of human observers or even better (Khan et al., 2020b). They can also extract useful representations for unlabeled images (unsupervised learning). More recently, they can also be learned effectively through the reinforcement learning method (Arulkumaran et al., 2017) and federated learning (Deng et al., 2022). For example, in the review paper (Wang C. et al., 2022) the authors analyzed the CNN use throughout the fresh fruit production chain and evaluation: flowering, growth, and picking (using ground or aerial platforms). Another important aspect is the fact that modern NNs are pre-trained, for example on the ImageNet (Deng et al., 2009) and PASCAL VOC (Everingham et al., 2015) databases, making the transition to the desired concrete application much easier and faster, with fewer training images.

The use of NNs involves three distinct phases: training, validation, and testing. The images from the available data set (including those obtained by augmentation) must be randomly divided between these three phases. The proportion is 70% - training, 20% - validation, and 10% - testing. The validation phase is used in some works to establish network confidence levels for collective intelligence (Popescu et al., 2022a) or decision fusion systems (Ichim and Popescu, 2020). Sometimes the testing phase is abandoned and then the proportion is 80% - training and 20% - validation.

### 4.2 Software used

Different software libraries and modules (most of them free) are used for image processing in successive tasks like obtaining orthomosaic, georeferenced maps, 3D models, machine learning, image annotation, implementing deep neural networks, etc. To obtain useful information for tree canopy extraction and segmentation, the images acquired by UAVs must be processed with various software, for example, Agisoft Photoscan (https://www.agisoft.com/) to generate geo-referenced ortho-images (Kerkech et al., 2020; Adhikari et al., 2021). To implement the NN models the most used software and platforms were TensorFlow (https://www.tensorflow.org/), PyTorch (https://pytorch.org), and Keras (https://keras.io/). An important step in the learning and testing phases is image annotation. There is different software as image annotator like YOLOLabel for the YOLO series (Iqbal et al., 2021; Yuan and Choi, 2021) and VGG Image Annotator (Biffi et al., 2021).

### 4.3 Datasets

The databases used in the analyzed papers are divided into two groups: a) databases for learning/validating/testing NNs for the detection/classification/segmentation of objects of interest from the images acquired in the orchard and b) databases for configuring flights of photogrammetry or inside the orchards to collect data (images).

A pertinent presentation of public image databases for use in precision agriculture is made in (Lu and Young, 2020) which contains 34 such databases. Of these, 11 refer to orchards: DeepFruits, Orchard fruit, Date fruit, KFuji RGB-DS, MangoNet, MangoYOLO, WSU apple, Fuji-SfM, LFuji-air, MinneApple, and Apple Trees. They are created manually or by ground vehicles. Most are based on RGB images. Many times, augmentation, annotation, and sharing operations can be performed on the images from the databases when used in NNs. The augmentation operations, often necessary in the learning stage to establish the most correct parameters and weights, are not used in the validation or testing stages. To obtain correct training of NN sometimes the data set must carefully filter because it can contain errors. For example, the IP 102 dataset (Wu et al., 2019), with more than 75,000 images for pest detection, was filtered to obtain better results. The filtered dataset, HQIP102, containing 47,393 images of 102 pest classes on eight crops was used (Peng et al., 2023) to train and test NN for pest detection.

To be sure that the trained NNs will learn the main characteristics of the objects to be detected or classified and will be more robust in a natural environment such as the orchard, many researchers have performed data augmentation starting from the original data. For example, 15 different augmentation methods are mentioned in (Lei et al., 2022), such as Gaussian noise, impulse noise, out-of-focus blur, motion blur, zoom blur, elastic transformation, rotation transformation, random erase, random crop, random flip, fog, brighten, contrast, color dithering, and pixelated. To obtain good results on NN training, the classes in the dataset need to be balanced and annotated. In the case of data imbalance, the authors (Peng et al., 2023) proposed an efficient data augmentation based on a dynamic method that depends on the initial number of elements in each class. In addition to these classic augmentation operations, synthetic augmentation operations using NNs for generating new images such as GAN are also used lately (Lu and Young, 2020).

The applications studied through this manuscript often require large datasets for the training/validation of NNs. Unfortunately, these resources are not always well-defined or are restricted. There are also some exceptions such as (Torres-Sánchez et al., 2018) which list several point cloud collections.

The advantages of automatic analysis and labeling from UAV images are particularly important (Zortea et al., 2018): one day for automatic image labeling compared to one month for manual labeling in the field with a GPS locator and one week for manual labeling of images obtained from a UAV flight. To label manually, efficient software assisting tools were developed like labelImg used for annotation in the MangoYOLO dataset and VIA (VGG Image Annotator) used for annotation in the MinneApple dataset. Most datasets are created for image processing, classification, and segmentation inside the orchard with machine learning tools, but there are also datasets for photogrammetry applications, for example, the ODMdata page (https://github.com/OpenDroneMap/ODMdata) which contains a large collection of various data sets with open access (orchards, forest areas, parks, etc.).

It is worth mentioning that most identified databases deal with photogrammetry applications or, at most, with production estimation (fruit counting). In other words, there are no UAV collections that provide close-up images (to identify visually small bugs or morphology changes at the leaf level). In most papers, own data sets, specific to the application, were used, but there are also papers that were limited to public databases (**Table 3**).

**Table 3:** Public datasets used.

Dataset name	Characteristics	Year	Number of images	Link	References
**COCO-Stuff**	Contains pixel-level annotations of classes such as grass, leaves, tree, and flowers	2017	123,287 images, 886,284 instances	https://cocodataset.org/#download	(Caesar et al., 2018; Dias et al., 2018)
**AppleA, AppleB,**	Datasets containing apples, peaches, and pears	2018	207 images	https://data.nal.usda.gov/dataset	(Dias et al., 2018; Dias et al., 2018)
**MinneApple**	Benchmark dataset for apple detection, segmentation, and counting in the orchard	2019	1,000 images with 40,000 annotated objects	https://rsn.umn.edu/downloads	(Häni, 2020)
**IP102**	Contains 102 pest classes on eight crops.	2019	more than 75,000 images	https://www.kaggle.com/datasets/rtlmhjbn/ip02-dataset	(Wu et al., 2019), (Peng et al., 2023)
**Mango** **YOLO**	Image dataset acquired with a farm terrestrial vehicle for train, testing, and validation	2019	1730 images	https://figshare.com/articles/dataset/MangoYOLO_data_set/13450661/2	(Koirala et al., 2019a)
**Mendeley Data (dataset added)**	Image dataset acquired from a UAV over an experimental site; added to Mendeley	2020	314 images	https://data.mendeley.com	(Encinas-Lara et al., 2020)
**Pistachio** **Dataset**	Pistachio orchard with two different nadir angles	2021	248 images	https://doi.org/10.5281/zenodo.7271542	(Vélez et al., 2022)

### 4.4 Statistic performance indicators

Considering the results obtained from the experiments, the analyzed papers used the following elements that make up the confusion matrix (error matrix): true positive cases (TP), true negative (TN), false positive (FP), and false-negative (FN). Based on them, a series of statistical quality indicators were calculated for the assessment of detection, classification, or segmentation operations: Specificity (SPE), Sensitivity (SEN), Precision (PRE), Accuracy (ACC), Dice coefficient (F1 score) (DSC or F1), and Jaccard index (**Table 4**). If the application refers to several classes, many authors prefer to provide average values for DSC and ACC in all classes.

**Table 4:** Statistic performance indicators used in the review.

Indicator	Formula	Indicator	Formula
**▪**Specificity	$SPE=\frac{TN}{TN+FP}$	**▪**Sensitivity (Recall)	$SEN=\frac{TP}{TP+FN}$
**▪**Precision	$PRE=\frac{TP}{TP+FP}$	**▪**Accuracy	$ACC=\frac{TP+TN}{TP+TN+FP+FN}$
**▪**Dice coefficient (*F*1-score or simple *F*)	$DSC=\frac{2\cdot TP}{2\cdot TP+FP+FN}$	**▪**Jaccard index (In confusion matrices)	$J=\frac{TP}{TP+FN+FP}$
**▪**Intersection over Union or Jaccard index	$J\left(A,B\right)=IoU=\frac{\left|A\cap^{}B\right|}{\left|A\cup^{}B\right|}$	**▪**Mean Average Precision	$mAP=\frac{1}{n}\sum_{i=1}^{n}AP_{i}$
**▪**Coefficient of determination (R- squared)	$R^{2}=1-\frac{SSE}{SST}$	**▪**Capturing rate (*CR*)	$CR=\frac{captured\;objects}{real\;objects}$
**▪**Detection rate (*DR*)	$DR=\frac{detected\;objects\;}{captured\;objects\;}$	**▪**Statistical rate (*SR*)	$SR=\frac{detected\;objects}{real\;objects}$

In addition to these indicators, Intersection over Union or Jaccard index (IoU) was used to assess detection and segmentation. Mean Average Precision (mAP) is a statistical indicator used to evaluate the performance of NN for object detection. It is calculated as an average over the number of classes n of APi entities that represented the average detection accuracy for class i (**Table 4**). The mAP is calculated for different IoU thresholds. In the case of evaluating the correctness of the detection and counting of several objects in the image (for example, in the case of instance segmentation), some papers used Capturing Rate (CR), Detection Rate (DR), and Statistical Rate (SR) calculated based on the actual number of objects, the number of objects in the image and the number of objects detected by the computing system in the same image. Another indicator worth mentioning is the Coefficient of determination (R- squared), calculated from the sum of squares of residuals (SSE) and the total sum of squares (SST).

Also, learning time and operating time are considered. These time indicators strongly depend on the networks, the hardware used (CPU, GPU, computer cluster, etc.), the resolution, and the number of images.

### 4.5 New trends in the implementation of neural networks for orchard monitoring

The novelties of the recent papers in the analyzed field refer to the combination of several networks into decision systems to obtain better performances than the component networks, including a CNN as the backbone in other CNN (network in a network), the improvement (adaptation) of some networks for the respective application - hence the name of the network, and the improvement of well-established high-performance networks. The new trends in the use of NNs in orchard monitoring follow the general line regarding either the improvement of existing networks by optimizing resources and improving performance or by combining several NNs in network ensemble models. In this case, it can be noted either the decision of the global system through the majority vote of the decisions of the individual networks or through the weighted summation of the detection (or classification) probabilities offered by each component network of the ensemble. The weight of a network is assigned proportionally to its performance. To select the best NNs relative to an application, some papers present comparisons regarding the values of the performance indicators of several top NNs. Thus in (Torres-Sanchez et al., 2020) SegNet, U-Net, FC-DenseNet, DeepLabv+ Xception, and DeepLabv3+ MobileNetV2 are compared regarding tree segmentation from UAV images. The obtained performances by (Zhang and Zhang, 2023) were ACC: 88.9 – 96.7%, F1-score: 87 – 96.1%, and IoU: 77.1 – 92.5%. These networks can be combined into ensemble systems for better detection (Deng et al., 2021; Popescu et al., 2022a).

For areas with several orchards and different conditions, for unitary management regarding several diseases and insect pests, the authors (Deng et al., 2022) proposed a federated learning method of NNs from several sources (obviously, several UAVs). In this way, if an orchard has unbalanced or insufficient data for a disease/pest, then the data is compensated from the other orchards, resulting in better learning. For example, the improved Faster R-CNN model by (Deng et al., 2022) can recognize fruit diseases and insect pests under occlusion.

The popular networks were modified to improve their performances. In (Zhang and Zhang, 2023) an improved U-Net, namely MU-Net was implemented to segment the plant diseased leaf. A residual block (Resblock) and a residual path (Respath) were introduced into U-Net to overcome gradient problems and, respectively, to improve the feature information between the two paths of U-Net. For better performances on pest classification, DensNet 121 was improved (Peng et al., 2023) in three directions: input information feature, channel attention technique, and adaptive activation function. Each improvement creates a modified DensNet 121 model. The three models are combined into an ensemble and the final decision is based on the sum of the normalized confidence values for each pest category on these three NNs.

By simultaneously considering RGB and NIR images, more precise information can be obtained about the health of plants, including orchards or vineyards. For example, in (Kerkech et al., 2020) multimodal images (visible and infrared) are used for disease detection in grapevine crops. Patches of 360 × 480 pixels were cropped and analyzed from the original images (4608 × 3456 pixels). Two channels are selected green and NIR and the regions of interest are segmented on both channels. For the dataset, semi-automatic labeling was used in two steps: LeNet 5 and manual correction. Four classes are considered: shadow, ground, healthy, and symptomatic vine. Two SegNet models were evaluated and tested for segmentation in RGB and NIR channels. The symptomatic cases are interpreted considering the fusion by intersection and union of segmentations obtained by the two networks. The recommendation is to consider a system with more NNs.

Some common NNs were adapted for a specific application and got the name of the application: Vine Disease Detection Network (VddNet) (Kerkech et al., 2020), YOLO designed for mango fruit detection (MangoYOLO) (Koirala et al., 2021), network to detect the invasion degree of Solanum rostratum Dunal (DeepSolanum-Net) (Wang et al., 2021).

**Figure 6:**
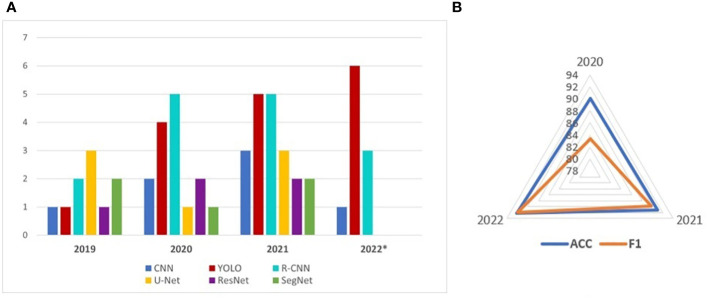
The most used NNs in orchards **(A)** and main performance indicators **(B)**.

A synthesis of the new trends of UAVs and NNs in the orchard monitoring context between 2020 and 2022 is done in **Table 5**. The trend of most used NNs as number of appearances in research papers between 2019–2022 were represented in **Figure 6A**. The symbol * marks the fact that at the time of writing the article, the Web of Science indexing for the year 2022 has not finished. An average of the main performance indicators is represented by the graph in **Figure 6B**. It can see that both ACC and F1 have an increasing trend, which means obtaining better-performing solutions.

**Table 5:** **A** summary of new trends for the orchard-UAV-NN triplet.

Model Novelty	Characteristics, Pros, and Cons	NN used and function	Performance indicators	References
**▪**Combining two different CNNs	**▪**Semantic segmentation of vegetation. **▪**Pros: Good results in a wetland mapping application. **▪**Cons: Slower training process.	**▪**SegNet with VGG16 **▪**SegNet with ResNet50 **▪**UNet with VGG16 **▪**UNet with ResNet50	**▪**ACC = 91% for SegNet with ResNet50 **▪**Time for NN training: 700 min	(Bhatnagar et al., 2020)
**▪**Fusing the outputs of two CNN, one for RGB and the other for NIR images	**▪**Two camera sensors for RGB and NIR. Disease detection in vine crops using segmentation **▪**Pros: Fusion by intersection is better than classes detected in the visible or infrared range: **▪**Cons: Reduced performances on segmentation due to the small training set and too few NNs in the system, long runtime	**▪**Two SegNet (RGB and NIR) **▪**Two LeNet5 (RGB and NIR) for pre-labeling	**▪**Leaf-level average ACC: 82.20% - fusion AND; 90.23% - fusion OR; **▪**Grapevine-level average ACC: 88.14% - fusion AND; 95.02% - fusion OR;	(Kerkech et al., 2020)
**▪**Net with a specific name for the application: DeepSolanum-	**▪**Segmentation of UAV images to detect the invasion degree of “Solanum rostratum Dunal” **▪**Pros: Reduced training time and complexity **▪**Cons: Performances must be improved	**▪**DeepSolanum-Net based on U-Net	**▪**Precision = 89.95% **▪**Recall = 90.3% **▪**IoU = 82.76% **▪**F1-score = 89.85%	(Wang et al., 2021)
**▪**Different CNN combined in a system for orchard monitoring **▪**Net with a specific name: MangoYOLO	**▪**Detect and count the fruits within images. Input: tree image. Output: total fruits per tree **▪**Pros: Good performance for fruit counting in one season. **▪**Cons: It is not a robust model in different seasons.	**▪**Multi Layered Perceptron (MLP), **▪**MangoYOLO model, **▪**Xception_count model with a regression block, **▪**Xception_classification model	**▪**Best R^2 =^ 94%	(Koirala et al., 2021)
**▪**Including a CNN as a backbone in other CNN	**▪**Detection and semantic segmentation of coconut trees **▪**Pros: Good ACC **▪**Cons: Need to classify and locate different kinds of trees.	**▪**Mask R-CNN with ResNet 101 as a backbone	**▪**mAP = 91% **▪**ACC (classification) = 97%	(Iqbal et al., 2021)
**▪**Dual network-based system to eliminate successively some FN and FP errors	**▪**Detecting and classifying harmful insects in orchards (HH) **▪** Pros: Good performance to detect insects in the foreground. **▪** Cons: Need to detect insects in a distant plane.	**▪**YOLOv.4 with DarkNet combined with EfficientNet B3	**▪**ACC = 95% **▪**F1-score = 92%	(Popescu et al., 2022b)
**▪**Combining NN YOLOv5s, DeepLabv3+ MobileNetv2	**▪**Detecting and segmentation of the logan fruit branch for logan harvesting using RGB-D camera **▪**Pros: Reduced operating time and good ACC semantic segmentation **▪**Cons: Limitations of object detection and segmentation in environmental interference conditions	**▪**Improved YOLOv5s for detection and DeepLabv3+ MobileNetv2 for semantic segmentation	**▪**ACC = 85.50% (fruit branch detection) **▪**ACC = 94.52% (fruit branch semantic segmentation)	(Li, D. et al., 2022)
**▪**Faster R-CNN improved with the Feature Pyramid Networks (FPN)	**▪**Count the number of pecans in an orchard **▪**Pros: Good mAP to identify pecans **▪**Cons: Influence of lighting on fruit recognition and detection.	**▪**Faster R-CNN and FPN	**▪**mAP = 95.932%	(Hu et al., 2022)
**▪**Federated learning (FL) and improved Faster R-CNN.	**▪**Multiple pest detection **▪**Pros: Can detect multiple pests in a short time. **▪**Cons: ACC must be improved	**▪**Faster RCNN with ResNet 101 and with FL	**▪**mAP = 89.34% **▪**ACC = 90.27% **▪**Detection time = 0.05 s	(Deng et al., 2022)
**▪**Combining three improved DensNet 121	**▪**Pest detection from an augmented big dataset **▪**Pros: Detecting pests on various agricultural crops **▪**Cons: Performances must be improved	**▪**Improved three DensNet 121 and combined them into a decision fusion system	**▪**ACC = 75.28%	(Peng et al., 2023)

## 5 Applications

In recent years, more and more tasks related to the monitoring of orchards in large areas are solved by the intelligent processing of data, and especially of images, collected with the help of drones. Most applications related to the use of UAVs and NNs in orchard monitoring refer to orchard mapping, pest and harmful insect detection, fruit detection, yield estimation, and orchard condition. In an automatic inspection of the orchard, for the desired application, the appropriate trajectory of the UAV must be specified and designed, according to Section 3. A major element in orchard surveillance is identifying regions or objects of interest. This may be at the macro level (orchard, tree lines, boundaries), medium level (corona shape estimation, tree center, and height identification), or micro level (counting fruits, pest detection, or insect detection). As expected, there is a large variety of approaches and tools to solve such problems. For example, (Torres-Sánchez et al., 2018) discusses canopy area, tree height, and crown volume. Noteworthy, the crown shape may vary even for the same type of tree (as remarked by (Mu et al., 2018) for peach orchards). Common geometric shapes considered for the crown shape are the cone, hemisphere, and ovoid (Torres-Sánchez et al., 2018). The precision of the estimation varies and strongly depends on the flight characteristics and camera performance (Gallardo-Salazar and Pompa-García, 2020).

As was mentioned in Section 4, there are cases where the networks take the name of the specific application. For example, the authors (Kestur et al., 2019) proposed a deep convolutional neural network architecture for mango detection using semantic segmentation named MangoNet. Also, the authors (Koirala et al., 2021) call the network YOLO used MangoYOLO, and (Sun et al., 2023) named YOLOP the modified YOLO v5 for pear fruit detection. The authors (Kerkech et al., 2020) proposed a deep convolutional neural network architecture for vine disease detection named VddNet with a parallel architecture based on the VGG encoder.

In the case of orchard monitoring using UAVs and NNs, there are several essential applications such as the detection and segmentation of orchards and individual trees, the detection of tree diseases, the detection of harmful insects, the identification of fruits and the evaluation of production, or the development of the orchard.

### 5.1 Orchard and tree segmentation

The mapping and segmentation of the orchards as well as the trees inside was the subject of many research articles from the analyzed period. Crop tree detection, location, and counting are estimated by (Sarabia et al., 2020; Dyson et al., 2019; Lobo Torres et al., 2020; Modica et al., 2020) based on UAV flight multispectral cameras, and morphological image processing techniques. Using U-Net and RGB images, the authors (Schiefer et al., 2020) perform tree species segmentation.

There are multiple ways to identify individual trees (canopy segmentation) in an orchard/forested area. These vary with the particularities of the specific trees and range in complexity from simple box partitioning like in (Horton et al., 2017) to handling irregular shapes and intermingled branches as in (Cheng et al., 2020) tested for cherry and apple trees orchards. Classically, the Hough transform for feature extraction has been often used but with relatively weak performance. Better performance was observed when using a Gaussian Mixture Model (Cheng et al., 2020). A similar approach is followed in (Dong et al., 2020), again for irregular crown shapes but this time applied to apple and pear trees. Crown segmentation is sometimes only an intermediary step for detecting the row lines and then, tree centers along each of these lines. (Zortea et al., 2018) implements such a mechanism for citrus orchards, a high-density case. Simply comparing the digital surface and terrain models (DSM and DTM) may also be used, as in (Gallardo-Salazar and Pompa-García, 2020) to geolocate trees and delineate their crowns.

The tree detection and classification procedure apply not only to curated environments (such as orchards) but also to natural growths which are more irregular in both tree size and placement like large boreal forest areas (Nevalainen et al., 2017). Another exception is (Tu et al., 2020) where high-resolution images were acquired from UAVs in a more complex context (areas with urban vegetation). The application is the semantic segmentation of trees of a specified species (Dipteryx alata - cumbaru class) using state-of-the-art networks. The NNs investigated were SegNet, U-Net, FC-DenseNet, and two DeepLabv3+ implementations (Xception and MobileNetV2) all with the same learning rates and optimizer for the learning phase. Moreover, a fully connected CRF (conditional random field) approach is proposed as a postprocessing step of the individual output NN decision. The results of using CRF were statistical performance improvement (ACC: 0.2% - 1.7%, F1-score: 0.2% - 1.9%, and IoU: 0.4% - 3%) and a decrease in computational efficiency (34.5 s for inference time). Regarding the performances of the studied networks, the best ACC, F1-score, and IoU (96.7%, 96.1%, and 92.5%) were obtained for FC-DenseNet and the lowest for DeepLabv3+Xception (88.9%, 87.1%, and 77.1%). Also, the best results for inference time were for FC-DenseNet (1.14 s) and the lowest for DeepLabv3+Xception (4.44 s).

It should be mentioned that some sources of error are systematic. For example, using a point cloud to estimate tree height naturally will provide less reliable height estimates if the tree shape narrows toward the top, which means that fewer points in the cloud are available for the 3D reconstruction (Gallardo-Salazar and Pompa-García, 2020). Even for simple photogrammetry applications, there are many features that may be considered. Beyond the standard segment length, segment intra-distance, and turn radius (the latter relevant only for fixed-wing UAVs) we may also consider height variation from segment to segment. E.g., in (Duarte et al., 2020) the segments follow the curvature of the terrain, leading to pictures taken along a surface that maintains a mostly constant height from the hilly ground beneath the camera. (Hulens et al., 2017) aims to detect through image processing the start and end points of an orchard row while traveling within it.

**Figure 7:**
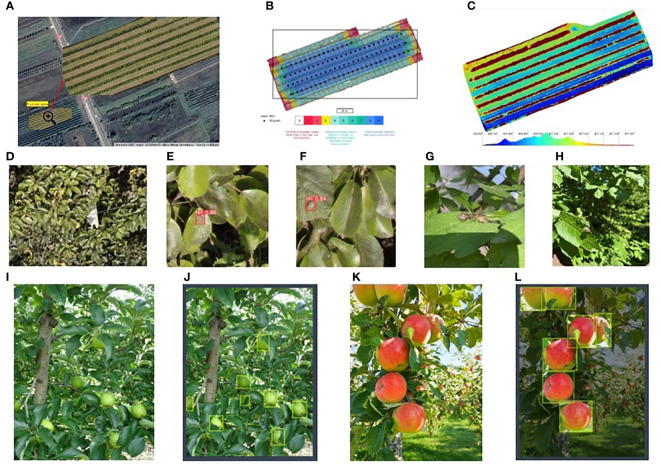
**(A)** Creating the elevation map: studied area, **(B)** Photo capture points, **(C)** Elevation map, **(D)** image from UAV containing HH in orchard, distant plan (4 m), **(E, F)** HH detected from D using image crops, **(G, H)** HH nymph at 0.6 m with manual acquisition, **(I)** Image with green apples in orchard, **(J)** Marked green apples, **(K)** Image with red apples in orchard, **(L)** Marked red apples.

To obtain useful information for tree canopy extraction and segmentation, the images acquired by UAVs must be processed with various software (for example, Agisoft Photoscan) to generate geo-referenced ortho-images (Apolo-Apolo et al., 2020a; Adhikari et al., 2021). For example, in **Figure 7** from a small, studied area the segmentation and elevation map is created using the photo capture points.

In most cases, the articles considered the detection and segmentation of some trees of a certain species, such as citrus (Csillik et al., 2018), palms (Culman et al., 2020), coconut (Iqbal et al., 2021), fig plant (Fuentes-Pacheco et al., 2019), etc., but the recommended solutions can also be applied to other types of orchards. In this case, the NNs system must be relearned with a new set of data (images) and the performances may be slightly different. Authors (García-Murillo et al., 2020) proposed the Cumulative Summation of Extended Maxima transform (SEMAX) methodology for the automatic individual detection of citrus and avocado trees.

A synthetic presentation of orchard and tree mapping and segmentation application is given in **Table 6**.

**Table 6:** Orchard and tree segmentation. Monitoring the evolution and condition of the orchard.

Purpose (orchard task)	Resources	Performance	References
Orchard and tree segmentation			
**▪**Detection of Citrus Trees based on a UAV flight and image processing in two steps: detection and classification	**▪**UAV; multispectral camera; Simple CNN for detection; Simple Linear Iterative Clustering algorithm (SLIC) for classification.	**▪**ACC=96.24%,	(Csillik et al., 2018)
**▪**Individual palms detection from high-resolution remote sensing images	**▪**UAV; RGB camera; RetinaNet	**▪**mAP=86.1%	(Culman et al., 2020)
**▪** Fig plant segmentation	**▪**UAV; RGB camera; encoder-decoder DCNN, inspired by SegNet architecture	**▪**ACC=93.85%	(Fuentes-Pacheco et al., 2019)
**▪**Tree detection and position	**▪**UAV; hyperspectral camera; different CNNs	**▪**F1 = 95.9%,	(Miyoshi et al., 2020)
**▪**Branch detection of apple trees	**▪**UAV; RGB camera; Pseudo-Color Images and Depth, R-CNN	**▪**REC=92%, **▪**ACC=86%	(Zhang et al., 2018)
**▪**Detection and segmentation of trunk/branch, apples, and leaves	**▪**Terrestrial platform; RGB-D camera; ResNet-18	**▪**ACC= 94.5%-94.8%	(Zhang, X. et al., 2019)
**▪**Identify the tree trunks and branches for a harvesting system	**▪**RGB camera; Deeplab v3+ with backbone: ResNet-18, VGG-16, and VGG-19	**▪**Per-class accuracy (PcA) =97%	(Zhang X. et al., 2021)
**▪**Semantic segmentation of citrus trees in a dense orchard	**▪**UAV; multispectral camera; FCN, U-Net, SegNet, DDCN, Deep-LabV3 +	**▪**ACC= 94.88%-95.96%	(Osco et al., 2021)
**▪**Detection and classification of individual tree	**▪**UAV; RGB camera; AlexNet, SqueezeNet, VGG 16; ResNet 50, DenseNet 121	**▪**ACC = 97.6% -99.5%	(Nguyen et al., 2021)
**▪**Dection and semantic segmentation of coconut trees	**▪**UAV; RGB camera; Mask R-CNN with ResNet101 as backbone	**▪**mAP=91%	(Iqbal et al., 2021)
**▪**Segmentation of planting rows of orange trees	**▪**UAV; RGB camera; Pipeline of two encoder-decoder networks (DetED – for detection and CorrED – for correction	**▪**ACC = 94% - 99.5%,	(Rosa et al., 2020)
Monitoring the evolution and condition of the orchard			
**▪**Evaluating the phenotypic characteristics of orange trees with influences on plant growth	**▪**UAV; multispectral camera; YOLO v3	**▪**PRE=99.9%	(Ampatzidis et al., 2020)
**▪**Evaluating the vigor and health of trees in a peach orchard using multispectral images	**▪**UAV; multispectral camera; Faster R-CNN	**▪**NA	(Cunha et al., 2021)
**▪**Recognition of spraying areas in the orchard.	**▪**UAV; RGB camera; improved Faster R-CNN	**▪**ACC=87.77% - 88.57%	(Khan et al., 2021)
**▪**Determination of the NDVI in a pomegranate orchard	**▪**UAV; Deep Stochastic Configuration Networks (DeepSCNs), regression model	**▪**R2 = 99.5%	(Niu et al., 2020)
**▪**Nitrogen concentration in an apple orchard	**▪**UAV; hyperspectral camera; backpropagation neural network (BPNN)	**▪**R2 = 77%	(Li, W. et al., 2022)
**▪**Nitrogen, Phosphorus, and Potassium foliar content retrieval in olive trees	**▪**UAV; multispectral camera; ANN	R2 = 63% - 95%	(Noguera et al., 2021)
**▪**Monitoring citrus orchards	**▪**UAV; RGB camera; FCRN-MTL	**▪**PRE=95%	(La Rosa et al., 2020)
**▪**Multispecies fruit flower (apple, peach, and pear) detection by semantic segmentation	**▪**Datasets publicly available; RGB camera; residual convolutional neural	**▪**F1 = 74.2%- 86%	(Dias et al., 2018)
**▪** Estimating olive tree’s biovolume	**▪**UAV; multispectral camera; Mask R-CNN based on ResNet50	**▪**F1 = 95%-98%	(Safonova et al., 2021)
**▪**Evaluating the temperature in an apple orchard for frost protection	**▪**UAV; RGB camera; thermal camera; YOLOv4	**▪**mAP= 66.08%-71.57%	(Yuan and Choi, 2021)

### 5.2 Monitoring the evolution and condition of the orchard

Most of cases, the conditions and evolution of an orchard are evaluated from multispectral images, as can be seen in **Table 6**. But, since NNs are implemented for RGB images (three color channels), for multispectral images less of these networks were used. There are exceptions presented in **Table 6**. For example, in (Cunha et al., 2021) the vigor and health of peach trees are evaluated using vegetable indexes like NDVI (normalized difference vegetation index), GNDVI (green NDVI), NDRE (normalized difference red edge index), and REGNDVI (red-edge GNDVI) calculated from multispectral images. Other research is focused on the detection of spraying areas (Khan et al., 2021) and concentrations of various chemical substances like Nitrogen, Phosphorus, and Potassium (Noguera et al., 2021) in the leaves. The summary of the orchard evolution monitoring is in **Table 6**.

### 5.3 Detection of pests and tree diseases in orchards

Pest detection using UAV is an important application of orchard monitoring because pests cause significant loss of crop production (Castrignanò et al., 2021). A recent review of the impact of climate change (IPPC Secretariat et al., 2021) on plant pests showed that pests have expanded to new areas. FAO estimates that every year the losses caused by pests are up to 40% of global crop production. Therefore, pests and disease detection and their spread prediction in real-time are needed for efficient and non-polluting interventions. Detecting the pests and diseases of trees in orchards as early as possible can limit their spread. Manual observation is timely loss and inefficient (Roosjen et al., 2020). Using UAVs and artificial intelligence in pest detection and evaluation, important progress can be observed (Peng et al., 2023). The low-altitude flight of UAVs is more effective than the ground diagnosis which is time-consuming and laborious on large area monitoring (Lan et al., 2020).

In organic orchards, it is particularly important to detect and monitor insects, especially harmful ones. For this, there are several ways such as direct visual inspection of farmers, land platforms, or drones. The last option is the most efficient because it can cover a relatively important area in a short time. In (Sorbelli et al., 2022), a method of sweeping individual trees from an orchard for the detection and evaluation of harmful insects (Halyomorpha Halys (HH)) is described. Four NNs were compared (Ichim et al., 2022) to highlight the best-performing network in HH detection. For this experiment, the result was DenseNet201. Note that HH or other harmful insects are at least an order of magnitude smaller than fruits like apples or pears, hence the problem of accurately detecting and counting them is even more challenging. The partial occlusion is challenging and the estimation of the abundance of these insects is a difficult problem. In **Figure 7** some examples of HH at different stages of evolution and other insects in images taken on different conditions confirm the difficulty of real detection of insects in trees from UAV. As can be seen, the image from UAV at a safe distance (in automatic surveillance) contains insects hard to be distinguished and the recommended action is to split the images in crops and then detect the insects with NN. If the insects are in the first plan or in the public dataset the task detection is easier (Xing et al., 2019).

A synthetic presentation of tree disease and pest detection is given in **Table 7**.

**Table 7:** Detection of pests and tree diseases. Prediction and evaluation of orchard production .

Purpose (orchard task)	Resources and discussions	Performance	References
Detection of pests and tree diseases			
Infected or diseased trees detection	**▪**UAV; Faster R-CNN and Mask R-CNN approaches and fusing their outputs	**▪**SEN=81.67%	(Barmpoutis et al., 2019)
Detection of the citrus bacterial canker in disease development stages on Sugar Belle leaves and immature fruit	**▪**UAV; hyperspectral camera; the neural network Radial Basis Function (RBF) and the K-nearest neighbor (KNN)	**▪**ACC= 94%-100%	(Abdulridha et al., 2019)
Identification of fruit tree pests (Tessaratoma papillosa)	**▪**UAV; RGB camera; Tiny-YOLOv3	**▪**mAP= 38.12%- 95.33%	(Chen, C.J. et al., 2021)
Detection of the degree of HLB (huanglongbing) infection on large-scale orchard citrus trees	**▪**UAV; multispectral camera; stacked autoencoder (SAE) neural network	**▪**ACC= 99.72%	(Deng et al., 2020)
	**▪**UAV; multispectral camera; autoencoder	**▪**ACC=97.28%,	(Lan et al., 2020)
Detection of diseases in vineyards	**▪**UAV; multispectral camera; LeNet-5, SegNet – single or combination	**▪**ACC=78.72%-95.02	(Kerkech et al., 2020)
	**▪**UAV; RGB camera; LeNet-5	**▪**ACC=95.8%	(Kerkech et al., 2018)
	**▪**UAV; RGB camera; CaffeNet	**▪**NA	(Bouroubi et al., 2018)
	**▪**UAV; multispectral camera; VddNet	**▪**ACC=93.72	(Kerkech et al., 2020)
Detection of the presence and behavior of the nematode pest in coffee crops	**▪**UAV; RGB camera; U-Net and PSPNet	**▪**F1 = 69%	(Oliveira et al., 2019)
Detection of black rot on grape leaves	**▪**UAV; RGB camera; YOLOv3 with SPP module	**▪**PRE=94.05%, SEN=93.26%	(Zhu et al., 2021)
Sick tree detection	**▪**UAV; RGB camera; different CNNs: Alexnet, Squeezenet, VGG 16; Resnet 50, Densenet 121	**▪**ACC=97.6% -99.5%	(Nguyen et al., 2021)
Bug detection (Halyomorpha Halys) in an orchard	**▪**UAV; RGB camera; processing (NN)	**▪**NA	(Sorbelli et al., 2022), (Ichim et al., 2022)
Insect detection, invasive species (Anolis carolinensis)	**▪**UAV, RGB camera; SSD-based model of DCNN	**▪**PRE=70%	(Aota et al., 2021)
Invasion degree of “Solanum rostratum Dunal” detection	**▪**UAV; RGB camera; DeepSolanum-Net based on U-Net	**▪**F1 = 89.85%	(Wang et al., 2021)
Prediction and evaluation of orchard production			
**▪**Method for semantic segmentation and instance segmentation of bayberry fruit.	**▪**Terrestrial platform; RGB camera; Multi-module convolutional neural network	**▪**AP = 75.5% -91.3%	(Lei et al., 2022)
**▪**Accurate monitoring of fruit quantity in apple orchards	**▪**UAV inside orchard; RGB camera; YOLO v5s	**▪**AP = 90.39%	(Wang S. et al., 2022)
**▪**Yield estimates in apple orchards. Detecting apples on individual trees.	**▪**UAV; RGB camera; R-CNN	**▪**R^2^ = 80% - 86%	(Apolo-Apolo et al., 2020a)
**▪**Detection, counting, and estimation of the size of citrus fruits on individual trees	**▪**UAV; RGB camera; Faster R-CNN	**▪**F1 = 89%	(Apolo-Apolo et al., 2020b)
**▪**Detection and location of longan fruits	**▪**UAV; RGB camera; MobileNet backbone used to improve YOLOv4	**▪**mAP = 54.22 -89.73%	(Li D. et al., 2021)
**▪**Holly fruits detection and counting	**▪**UAV; RGB camera; YOLOX	**▪**DR >99%	(Zhang Y. et al., 2022)
**▪**Canopy extraction. Detect mango and predict the number on the tree	**▪**Terrestrial platform; RGB camera; Mango YOLO, Xception, Random Forest	**▪**R^2^ = 98%	(Koirala et al., 2021)
**▪**Detect apple fruit in the orchard	**▪**Manual images; RGB camera; comparing RetinaNet, Libra-RCNN, Cascade-RCNN, Faster-RCNN, FSAF, HRNet, and ATSS	**▪**Maximum AP = 94.6%	(Biffi et al., 2021)
**▪**Longan harvesting UAVs. Branch detection and fruit branch semantic segmentation.	**▪**UAV; RGB-D camera; YOLOv5s – for detection, and improved DeepLabv3+ (MobileNet v2) for semantic segmentation	**▪**ACC = 85.50% – 94.52%	(Li D. et al., 2022)
**▪**Grape detection, instance segmentation	**▪**RGB camera; Mask R-CNN with ResNet 101 as the backbone	**▪**F1 = 91%	(Santos et al., 2020)
**▪**Pear (fruit) detection	**▪**RGB camera; YOLO-P	F1 = 96.1%	(Sun et al., 2023)

### 5.4 Prediction and evaluation of orchard production

As specified by (Wang C. et al., 2022; Koirala et al., 2019b) the evaluation of fruit production is an important activity both from the social and economic points of view. The authors used a combined YOLO5 and FlowNet2 scheme to improve apple detection in an orchard for accurate yield estimation. They claim a good performance and a framerate of 20 frames/second even for partially occluded targets and under varying illumination conditions. This is in contrast with typical applications where the analysis is carried out offline.

The standard, encountered in virtually all aerial systems older than 5-10 years, is to gather the raw data and, at most, do some preliminary preprocessing before sending it to a ground station for further analysis. This has the obvious benefit of minimizing the hardware complexity and energy requirements for the drone but makes impractical “live” implementations where the mission must be updated on-the-fly from the gathered information. Recent applications, due to significant hardware resources, have started to handle increasing parts of the workflow onto the drone. While the effort is by no means trivial, dedicated software such as Jetson Nano, Google Coral, and the like permit image processing directly onto the drone. This means that decisions may be taken in a fully local manner (without interaction with the ground). Even a supervisor (human or software agent) still must be in the loop (as is the case for most commercial applications), there still is the benefit of reduced bandwidth allocation (since more steps of the image processing are done on the platform, it means that only relevant information is exchanged with the ground).

On the other hand, for position correction, collision avoidance, and even target counting (Wang S. et al., 2022), optical flow methods which compare consecutive frames to detect changes are used. This has the advantage of improving performance but comes usually with a reduction in resolution (since video frames have, unavoidably, less resolution than static images).

The great majority of drone trajectories are out of a plane (images/videos are taken top-down while the drone is flying over the treetops). Still, there are some results such as in (Wang S. et al., 2022) where the drone travels mid-row, through the orchard’s rows.

Using artificial intelligence methods to process the images acquired by autonomous terrestrial or aerial platforms, the conditions for picking fruits that have reached maturity in the optimal period can be improved. This approach leads to increased economic efficiency for orchards (Lei et al., 2022). Fruit estimation is challenging and the number of fruits on a tree cannot be measured exactly due to occlusions (Zhang X. et al., 2019).

Because of the similarity between the fruit and the leaf, the detection of green citrus fruits or green apples (**Figure 7**) is quite difficult. The authors (Zheng et al., 2021) proposed a modification of the YOLO neural network modules (starting from YOLO v4), called YOLO BP which detects the respective fruits with higher precision than YOLO v4. If the fruits are a color different from the leaves or are not obturated the detection task is easier (**Figure 7**). NIR is used especially for highlighting the leaves and the production of almonds in a tree. For example, in (Tang et al., 2023) aerial multi-spectral images (near-infrared, red edge, red, and green) are processed by a CNN to estimate the almond production in an orchard with a coefficient of determination, R2 = 96%. It is specified that the sun-shadow effect can decrease system performance.

A synthetic presentation of fruit production evaluation is given in **Table 7**.

## 6 Discussion

The use of UAVs and NNs for image processing in orchard monitoring is a relatively new method open to both research and end-user implementation. This was possible due to the development of new technologies in recent years and the decrease in the prices of the necessary equipment. Unfortunately, most of the current UAV applications are relatively simple from the viewpoint of trajectory generation (straight lines or successive set points to be reached). Still, continuous advances in hardware capabilities and the expected expansion of mission complexity mean that more complex scenarios will be defined and tackled. Continuous reduction in size, cost, and dimensions means that various sensor mechanisms (Lidar for example) may now be mounted onboard. Not least, improvements in embedded image processing (software and hardware modules such as Jetson Nano or Google Coral) mean that image-based positioning is now increasingly used. Henceforth, we expect that algorithms initially tailored for ground vehicles will be adapted in the next few years to aerial systems. For example, a great many algorithms exist for in-lane orchard navigation for ground autonomous systems (small-sized tractors, (Emmi et al., 2021)) and it should be possible to adapt them with minimal modifications. Although it is preferable to other methods such as terrestrial platforms or human operators, automatic UAV flight and establishing the trajectory inside the orchard for the acquisition of images is sometimes a real challenge due to several aspects such as: a) keeping a safe distance from tree branches, b) obtaining a continuous 3D surface (similar to orthomosaic) from which to cut out the images to be analyzed, c) detecting, segmenting and classifying small (insects, some fruits, diseases) and/or partially covered objects, d) large differences in brightness, e) background difficulty, etc. All this, including the characteristics of public databases (if they are used) leads to different performances for the same type of application.

It can be noted that, in general, the performances obtained depend both on the networks used and on the quality of the acquired data set. Many times, the division of high-resolution acquired images into sub-images (patches) and their analysis by the proposed NNs give better results than the processing of large images through the resizing required by the networks. This solution can be useful when trying to detect small objects in trees (such as insects). The performance of networks or systems made of multiple networks leans either on meeting the needs of precision or on meeting the needs fast processing, or on the compromise between these two. Anyway, for a large-scale application, on various farms, a solution that saves resources or a remote processing solution *via* the Internet is preferable. Another recommendation is to use, in situations where NIR images provide relevant information, to combine NNs for RGB with NNs for NIR in a global decision system.

There are several review articles with the topic of some common parts with this article, but none that include the triplet orchard, UAV, and NNs. Their descriptions and the novelty introduced in our paper are presented in **Table 8**.

**Table 8:** Recent review/survey papers on similar topics.

Paper	Description	Period	Ref.	Our differences (improvement or novelty)
(Kamilaris and Prenafeta-Boldú, 2018)	**▪**Using CNNs in agriculture. **▪**Comparing NN with other techniques in agricultural applications, high precision, and accuracy are obtained.	1995-2018	62	**▪**Focused on orchard monitoring from different points of view (applications). Focused on new trends in NN usage. Graphs on the evolution of UAV and NN use in the last period. Description of using UAVs for image acquisition. More references. New period.
(Koirala et al., 2019b)	**▪**Using DL for fruit detection and yield estimation. **▪**Comparing the statistical performances of CNN methods.	1991-2019	83	**▪**Focused on orchard monitoring from different points of view. Focused on new trends in NN usage. Graphs on the evolution of UAV and NN use in the last period. Description of using UAVs for image acquisition. More references. New period.
(Barbedo, 2019)	**▪**Using UAVs and image acquisition and processing to monitor and assess the plant stresses.	2003-2018	169	**▪**Focused on orchard monitoring from different points of view (applications). Focused on new trends in NN usage. Graphs on the evolution of UAV and NN use in the last period. More references. New period.
(Ma et al., 2019)	**▪**Using deep NNs in general remote sensing applications.	1991-2018	148	**▪**Focused on orchard monitoring from different points of view. Focused on new trends in NN usage. Graphs on the evolution of UAV and NN use in the last period. Description of using UAVs for image acquisition. More references. New period.
(Iost Filho et al., 2020)	**▪**Using multi-copters in pest management to identify harmful areas and to accurately spray pesticides. Sensing and actuation UAVs are investigated in agricultural systems	1986-2019	320	**▪**Focused on orchard monitoring from different points of view (applications). Focused on detailed descriptions of NN used and new trends. Graphs on the evolution of UAV and NN use in the last period. New period.
(Lu and Young, 2020)	**▪**Analyzing and establishing the main characteristics of 34 public image DSs for computer vision tasks in precision agriculture: 15 on weed control, 10 on fruit detection, and 9 for other applications.	2009-2020	98	**▪**Focused on orchard monitoring from different points of view (applications). Focused on new trends in NN usage. Description of using UAVs for image acquisition. Graphs on the evolution of UAV and NN use in the last period. More references. New period.
(Naranjo-Torres et al., 2020)	**▪**Using CNN for fruit recognition. Presentation of fundamentals, tools, and examples of CNNs for fruit sorting and quality control.	1998-2020	104	**▪**Focused on orchard monitoring from different points of view. Focused on new trends in NN usage. Description of using UAVs for image acquisition. Graphs on the evolution of UAV and NN use in the last period. More references. New period.
(Zhang et al., 2020)	**▪**Using DL for dense scenes analysis in agriculture. Analyzing the challenges in dense agricultural scenes. Presentation of architectures of DL algorithms and CNNs used in dense agricultural scenes	1988-2019	122	**▪**Focused on orchard monitoring from different points of view (applications). Focused on new trends in NN usage. Graphs on the evolution of UAV and NN use in the last period. Description of using UAVs for image acquisition. More references. New period.
(Dhaka et al., 2021)	**▪**Using DCNN for prediction of plant diseases from leaf images.	1989-2021	124	**▪**Focused on orchard monitoring from different points of view (applications). Description of using UAVs for image acquisition. Graphs on the evolution of UAV and NN use. More references.
(Li L. et al., 2021)	**▪**Using DL for plant leaf disease detection and classification	2006-2020	113	**▪**Focused on orchard monitoring from different points of view (applications). Description of using UAVs for image acquisition. Graphs on the evolution of UAV and NN use. More references.
(Liu and Wang, 2021)	**▪**Using DL for plant diseases and pest detection, considering three functions of NN: classification, detection, and segmentation.	2006-2021	108	**▪**Focused on orchard monitoring from different points of view (applications). Description of using UAVs for image acquisition. Graphs on the evolution of UAV and NN use. More references.
(Olson and Anderson, 2021)	**▪**Presentation of UAVs, image sensors, image acquisition, image processing, and their applications in agriculture	1973-2021	154	**▪**Focused on orchard monitoring from different points of view (applications). Focused on new trends in NN usage. Description of using UAVs for image acquisition. Graphs on the evolution of UAV and NN use in the last period. More references.
(Zhang C. et al., 2021)	**▪**Presentation of orchard management with small UAVs	1978-2019	147	**▪**Focused on new trends in NN usage for image processing for orchard monitoring. Graphs on the evolution of NN use in the last period. More references. New period.
(de Castro et al., 2021)	**▪**Using UAVs for vegetation monitoring considering diverse agricultural and forestry scenarios such as vegetation indices, technological goals, and applications.	2004-2021	48	**▪**Focused on orchard monitoring from different points of view (applications). Focused on detailed descriptions of NN used and new trends. Graphs on the evolution of UAV and NN use. More references.
(Wang C. et al., 2022)	**▪**Detecting the phases of fruit evolution from flower, growth, ripening, picking, and classification, based on the analysis of images captured by terrestrial or aerial robots. NNs with one or two stages, built for object detection were considered.	1986- 2022	201	**▪**Focused on orchard monitoring from different points of view (applications). More NNs. Focused on new trends in NN usage. Description of using UAVs for image acquisition. Graphs on the evolution of UAV and NN use in the last period. More applications

## 7 Conclusions

This review covers a critical gap in modern orchard monitoring considering the essential contribution of both UAV and NNs as exponents of new technologies. As can be seen both from the analysis of research articles and review articles, only in recent years have these hardware/software resources been involved and analyzed in research in the field. Both the advantages offered by the two components (UAV and NN) of the analyzed orchard monitoring systems were highlighted as well as the challenges due to the difficulties encountered in real orchards, related to the UAV flight inside the orchards among the trees and the detection of small objects such as fruits or insects inside the crowns. The newest technologies used in modern orchards were analyzed in support of increasing production, increasing fruit quality, and eliminating pests and diseases through environmentally friendly means. Special emphasis was placed on the new trends in the development of the main analyzed vectors, namely NNs, and UAVs. The final discussion regarding the comparison with other review articles highlights the article’s contributions regarding improvements and new approaches. We hope the paper will help the researchers and producers of modern systems for orchard monitoring in the context of Agriculture 4.0. As previously stated in the paper, a limitation of the approach is the relatively small number of existing research articles in the complex topic of orchard monitoring-UAV-neural networks (it is a new field, in full expansion). As a future direction, we will follow the ever-growing evolution in this field, based on the fusion of information from terrestrial and aerial robots, for the most efficient monitoring of orchards using artificial intelligence techniques.

## Author contributions

Conception: DP, LI. Project administration: DP, LI, and FS. Writing – original draft: DP, LI, and FS. All authors contributed to the article and approved the submitted version.

## Glossary

**Table:**

AKAZE	Accelerated-KAZE
ACC	Accuracy
AI	Artificial Intelligence
AP	Average Precision
ATSS	Adaptive Training Sample Selection
CRF	Conditional Random Field
CNN	Convolutional Neural Network
CPU	Central Processing Unit
CR	Capturing rate
DASNet	Dual Attentive fully convolutional Siamese Network
DB	Database
DCNN	Deep Convolutional Neural Network
DDCN	Dynamic Dilated Convolution Network
DeepSCN	Deep Stochastic Configuration Network
DL	Deep Learning
DR	Detection Rate
DS	Dataset
DSC	Dice Coefficient
DSM	Digital Surface Model
DTM	Digital Terrain Model
F1	Dice Coefficient (F1 Measure)
FCN	Fully Convolutional Network
FCRN	Fully Convolutional Regression Network
FCRN-MTL	Fully Convolutional Regression Network Multi-Task Learning
FN	False Negative
FP	False Positive
FPN	Feature Pyramid Networks
FSAF	Feature Selective Anchor-Free
GDAL	Geospatial Data Abstraction Library
GIS	Geographic Information System
GNSS	Global Navigation Satellite System
GPS	Global Positioning System
GPU	Graphics Processing Unit
HRNet	High Resolution Network
IoT	Internet of Things
IoU	Intersection-Over-Union
KNN	K-Nearest Neighbor
mAP	Mean Average Precision
ML	Machine Learning
NDVI	Normalized Difference Vegetation Index
NIR	Near-infrared
NN	Artificial Neural Network
ODM	Open Drone Map
PRE	Precision
PSPNet	Pyramid Scene Parsing Network
RBF	Radial Basis Function
R-CNN	Region-Based CNN
ResNet	Residual Neural Network
RGB	Red-Green-Blue (images)
RTK	Real-Time Kinematic Positioning
RoI	Region of Interest
ROS	Robot Operating System
SAE	System Architecture Evolution
SAR	Synthetic-aperture radar
SegNet	Semantic Segmentation Network
SEN	Sensitivity
SPE	Specificity
SPP	Spatial Pyramid Pooling
SR	Statistical Rate
SSD	Single Shot MultiBox Detector
TN	True Negative
TP	True Positive
TSP	Traveling Salesman Problem
UAV	Unmanned Aerial Vehicle
UAS	Unmanned Aerial System
VGG	Visual Geometry Group
WOS	Web of Science
YOLO	You Only Look Once
